# Identification and Molecular Dissection of IMC32, a Conserved *Toxoplasma* Inner Membrane Complex Protein That Is Essential for Parasite Replication

**DOI:** 10.1128/mBio.03622-20

**Published:** 2021-02-16

**Authors:** Juan A. Torres, Rebecca R. Pasquarelli, Peter S. Back, Andy S. Moon, Peter J. Bradley

**Affiliations:** a Department of Microbiology, Immunology and Molecular Genetics, University of California, Los Angeles, Los Angeles, California, USA; b Molecular Biology Institute, University of California, Los Angeles, Los Angeles, California, USA; University of Pittsburgh

**Keywords:** inner membrane complex, *Toxoplasma gondii*, coiled-coil domain, endodyogeny, palmitoylation, parasitology

## Abstract

The inner membrane complex (IMC) is a unique organelle of apicomplexan parasites that plays critical roles in parasite motility, host cell invasion, and replication. Despite the common functions of the organelle, relatively few IMC proteins are conserved across the phylum and the precise roles of many IMC components remain to be characterized. Here, we identify a novel component of the Toxoplasma gondii IMC (IMC32) that localizes to the body portion of the IMC and is recruited to developing daughter buds early during endodyogeny. IMC32 is essential for parasite survival, as its conditional depletion results in a complete collapse of the IMC that is lethal to the parasite. We demonstrate that localization of IMC32 is dependent on both an N-terminal palmitoylation site and a series of C-terminal coiled-coil domains. Using deletion analyses and functional complementation, we show that two conserved regions within the C-terminal coiled-coil domains play critical roles in protein function during replication. Together, this work reveals an essential component of parasite replication that provides a novel target for therapeutic intervention of T. gondii and related apicomplexan parasites.

## INTRODUCTION

Toxoplasma gondii is a member of the phylum Apicomplexa, which includes many human pathogens, such as Plasmodium falciparum, the causative agent of malaria, and *Cryptosporidium* spp., a leading cause of diarrheal diseases (1–3). T. gondii is the most widespread member of the phylum, as it infects all mammals, including approximately one-third of the world’s human population (4). Most human infections are asymptomatic, but the parasite causes serious disease in immunocompromised individuals and congenitally infected neonates. Apicomplexan parasites share a number of unique organelles that are necessary for maintaining their intracellular lifestyles and causing disease (5). One of these organelles is the inner membrane complex (IMC), a peripheral membrane and cytoskeletal system that underlies the parasite’s plasma membrane and plays central roles in motility, invasion, and replication (6).

The IMC is composed of a series of flattened membrane vesicles, known as alveoli, which are sutured together like a quilt, as well as a supporting cytoskeletal network of intermediate filament-like proteins called alveolins (7–9). In T. gondii, the alveoli are organized into three rows of rectangular plates along with a single cone-shaped plate called the apical cap at the apical end of the parasites (10). Detergent fractionation demonstrates that most IMC proteins localize either to the alveolar membrane or the cytoskeletal network, though many network-associated proteins are likely also tethered to the membranes (9, 11, 12). While some IMC proteins, such as the GAPM proteins, IMC25, and GAP40, are embedded in the alveoli via transmembrane domains (13–15), the majority are tethered to the IMC membrane by myristoylation and/or palmitoylation, which are carried out via a cytoplasmic N-myristoyl transferase (NMT) or a target membrane palmitoyl acyltransferase (PAT), respectively (16, 17). Two essential IMC-localized PATs that are responsible for modifying a growing number of known palmitoylated IMC proteins were recently characterized (18–22). While many of the known acylated IMC membrane proteins are dispensable for parasite survival (e.g., ISP1-4 and HSP20), the precise identity and functions of essential palmitoylated proteins are still not well understood.

The IMC plays several crucial roles in the lytic cycle of T. gondii. The IMC houses the glideosome, the actin-myosin motor that provides the force necessary for gliding motility and host cell invasion (23–25). The apical cap portion of the IMC has recently been shown to play an important role in organizing the conoid, a microtubule basket-shaped structure that is critical for the secretion of the micronemes for host cell adhesion, and for the rhoptries, which are necessary for host cell penetration (26–28). Importantly, the IMC also serves as the scaffold for forming daughter buds during endodyogeny, the internal budding process of replication in T. gondii tachyzoites (6). Endodyogeny begins with the duplication and segregation of the centrosomes and kinetochores, followed by subpellicular microtubule assembly, IMC formation, and daughter cell budding. Newly synthesized or replicated organelles are packaged into the developing IMC scaffold until budding is complete, at which point the maternal IMC is disassembled and the daughter cells emerge, adopting the maternal plasma membrane (29). Although all apicomplexan parasites are dependent on the IMC for their various internal budding processes, many components of the IMC machinery are not conserved across the phylum (11, 12, 30, 31).

In addition to trafficking to the correct organellar subdomain, the timing of expression and recruitment of IMC proteins to the daughter buds during endodyogeny also likely plays an important role in IMC protein functions. Some of the earliest expressed proteins include the AC9/AC10/ERK7 complex, which is essential for invasion, and F-box protein TgFBXO1, which plays an important role in replication (26–28, 32). These are followed by ISP1 and ISP3, which are dispensable and recruited independently of the cortical microtubules which drive bud development (21, 33). Some proteins, such as the cytoskeletal alveolins IMC3 and IMC6, are recruited to daughter buds later in the replication process, and other IMC proteins are exclusively recruited to the maternal IMC, after endodyogeny is complete (IMC7, -12, and -14) (9, 34). Although formation of the IMC involves precisely timed recruitment of different IMC components to specific subdomains, the exact contribution of each of these components and the importance of correct timing remain poorly understood.

Here we report the discovery of a novel IMC protein (IMC32), which we identified from our previous *in vivo* biotinylation (BioID) experiment using the early daughter apical cap protein AC9 as the bait (27, 28). We show that IMC32 is an early daughter IMC protein and characterize its timing of recruitment relative to other IMC components. We also find that IMC32 is a membrane-associated IMC protein that resides in the body portion of developing daughter buds and that localization is dependent on a strongly predicted N-terminal palmitoylation site. Surprisingly, we find that proper protein trafficking also requires a series of conserved C-terminal coiled-coil (CC) domains. Using a conditional knockdown system, we demonstrate that IMC32 is essential for parasite survival and that its conditional depletion results in the complete collapse of the IMC. Finally, we use a series of truncations and internal deletions to dissect the coiled-coil domains to determine how these domains serve IMC32 function. These deletions show that two of the most conserved regions of the coiled-coils play essential roles in the ability of IMC32 to govern parasite replication. Taken together, we identify and characterize a novel IMC membrane protein that is absolutely essential for parasite replication.

## RESULTS

### IMC32 is an early daughter IMC protein.

**FIG 1:**
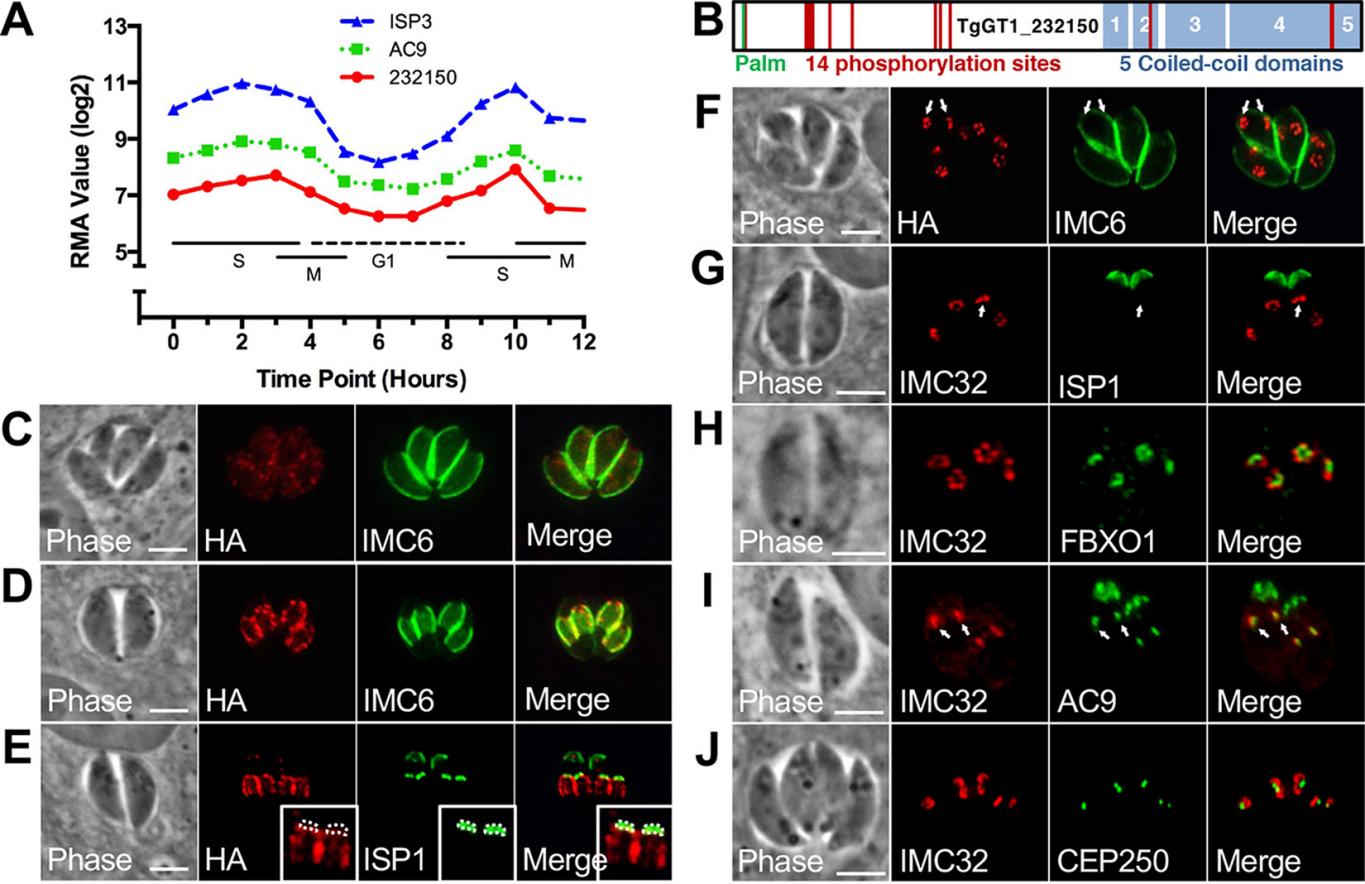
IMC32 is a daughter-enriched, membrane-bound IMC protein. (A) Cell cycle gene expression profile showing that TgGT1_232150 is similar to the known IMC proteins AC9 and ISP3. Robust multi-array average (RMA) is shown across the cell cycle (hours). (B) Gene model for TgGT1_232150 highlighting its predicted palmitoylation site, coiled-coil domains, and phosphorylation sites. (C) In nondividing parasites, IMC32 is difficult to detect by IFA, and its localization is diffuse in the cytoplasm. Red, mouse anti-HA; green, rabbit anti-IMC6. (D) TgGT1_232150 localizes to developing daughter buds, where it colocalizes with IMC6. Red, mouse anti-HA; green, rabbit anti-IMC6. (E) IMC32 is excluded from the apical cap region of the IMC. The apical cap is labeled with ISP1 and outlined with dotted lines in the inset. Red, rabbit anti-HA; green, mouse anti-ISP1. (F) IMC32 localizes to daughter buds (arrows) prior to the appearance of IMC6. Red, mouse anti-HA; green, rabbit anti-IMC6. (G) IMC32 is also recruited before the early daughter marker, ISP1. Arrows point to two daughter buds that contain IMC32 but lack ISP1. Red, rabbit anti-HA; green, mouse anti-ISP1. (H) IMC32 is recruited to earliest daughter buds at approximately the same time as 3×HA-tagged TgFBXO1. Red, mouse anti-Ty; green, rabbit anti-HA. (I) IMC32 is also recruited to the earliest daughter buds (arrows) similar to 3×HA-tagged AC9. Red, mouse anti-Ty; green, rabbit anti-HA. (J) IMC32 is recruited to daughter buds in close proximity to the centrosome marked by 3×HA-tagged CEP250, further indicating that it is a component of the daughter cell scaffold. Red, mouse anti-Ty; green, rabbit anti-HA. Scale bars for all images, 4 μm.

We previously generated a large data set of candidate IMC proteins from our BioID experiments using AC9 as the bait (28). Our top hits from this data set were filtered for proteins with cyclical expression patterns similar to known IMC proteins, negative genome-wide CRISPR screen (GWCS) scores, homology to known proteins, and potential acylations that may enable IMC membrane association (16, 35, 36). Using these criteria, TgGT1_232150 emerged as an interesting target for study, as it had a cyclical expression profile similar to AC9, as well as to ISP3, a daughter-enriched IMC protein that is palmitoylated and myristoylated (Fig. 1A) (21, 35). TgGT1_232150 also contains a strongly predicted N-terminal palmitoylation site at residue 7 and a −4.32 GWCS score (see Fig. S1A in the supplemental material) (36, 37). While the protein lacks homology to known proteins, other features include a series of 5 C-terminal coiled-coil domains and 14 phosphorylation sites identified by phosphoproteomics analyses (Fig. 1B; Fig. S1B and C) (38, 39). TgGT1_232150 appears to be conserved in the Apicomplexa, as orthologues are seen in *Neospora*, *Sarcocystis*, *Eimeria*, and *Cystoisospora*. In addition, BLAST analysis reveals a *Plasmodium* homologue, PF3D7_0717600, that has limited homology to TgGT1_232150 (2e^−14^) (Fig. S2). While we cannot determine if these are true orthologues or are homologous proteins, PF3D7_0717600 also contains predicted N-terminal palmitoylation sites (residues 7 and 13) and a similar series of C-terminal coiled-coil domains (Fig. S2A to C). Intriguingly, PF3D7_0717600 is also predicted to be very important or essential in *Plasmodium* by a genome-wide *piggyBac* transposon screen (40).

10.1128/mBio.03622-20.1 FIG S1 Predicted features of identified target, TgGT1_232150. (A) The predicted palmitoylation site scoring >12.0 by use of CSS Palm 4.0 (http://csspalm.biocuckoo.org) is shown (37). (B) The 14 phosphorylation sites predicted by phosphoproteomics analyses are listed (39, 45). (C) Coiled-coil domains predicted by the COILS server (38). Using a cutoff of 0.75, we sectioned the C-terminal cluster into five coiled-coil domains, labeled 1 to 5. Coiled-coil 1 (CC1) was designated from amino acids (aa) 522 to 549, CC2 from aa 561 to 645, CC3 from aa 665 to 738, CC4 from aa 743 to 918, and CC5 from aa 933 to 974. Download FIG S1, TIF file, 0.3 MB. Copyright © 2021 Torres et al. 2021 Torres et al. https://creativecommons.org/licenses/by/4.0/ This content is distributed under the terms of the Creative Commons Attribution 4.0 International license.

10.1128/mBio.03622-20.2 FIG S2 Predicted features of PF3D7_0717600, a TgGT1_232150 (IMC32) homologue in P. falciparum. (A) Alignment of TgGT1_232150 with PF3D7_0717600 was generated using Clustal Omega (66) and shaded using BoxShade (https://embnet.vital-it.ch/software/BOX_form.html). Black highlights indicate identity; gray highlights indicate similarity. The coiled-coil domains in TgGT1_232150 are labeled. (B) Predicted palmitoylation sites scoring >12.0 by use of CSS Palm 4.0 (http://csspalm.biocuckoo.org) are listed (37). (C) PF3D7_0717600 has a series of C-terminal coiled-coil domains, as predicted by the COILS server (38). Using a cutoff of 0.75, we sectioned the C-terminal cluster into three coiled-coil domains, labeled CC1 to -3. Coiled-coil 1 (CC1) was designated from residues 792 to 826, CC2 from residues 870 to 1005, and CC3 from residues 1014 to 1048. Download FIG S2, TIF file, 0.7 MB. Copyright © 2021 Torres et al. 2021 Torres et al. https://creativecommons.org/licenses/by/4.0/ This content is distributed under the terms of the Creative Commons Attribution 4.0 International license.

To determine the localization of TgGT1_232150, we used CRISPR/Cas9 to tag the endogenous locus with a C-terminal 3×HA epitope tag (41). In nondividing parasites, the protein was difficult to detect and appeared to be spread throughout the cytoplasm (Fig. 1C). In replicating parasites, TgGT1_232150 localizes to the body of parasite daughter buds, as assessed by colocalization with the IMC body alveolin IMC6 (Fig. 1D) (9). Costaining with the apical cap marker ISP1 showed that TgGT1_232150 is excluded from the apical cap portion of the organelle (Fig. 1E) (21). In mid to late daughters, the localization of TgGT1_232150 appeared spotty and seemed to form discontinuous longitudinal stripes along the body of the developing daughter buds. To ensure that this was not due simply to low detection, we used a spaghetti monster OLLAS tag (sm_OLLAS) to increase the strength of the signal (42). We also costained for the IMC suture protein ISC6 to determine if this pattern colocalized with the sutures that tether the alveolar plates of the organelle together (12). Tagging IMC32 with sm_OLLAS substantially increased the brightness of the signal but did not change the localization pattern. We could not observe a definitive colocalization with ISC6, which is predominantly seen as transverse stripes in the developing daughter buds (Fig. S3). Based on these data, we concluded that TgGT1_232150 is a daughter-enriched IMC body protein and named it IMC32.

10.1128/mBio.03622-20.3 FIG S3 Localization of IMC32 and ISC6 in developing daughter buds. IFA showing the localization of IMC32 and ISC6 in developing daughters. ISC6 predominantly stains the transverse sutures in daughter buds and does not definitively colocalize with IMC32. Red, rat anti-OLLAS (detecting sm_OLLAS-tagged IMC32); green, mouse anti-Ty (detecting 2×Strep3×Ty-tagged ISC6). Scale bar, 2 μm. Download FIG S3, TIF file, 0.3 MB. Copyright © 2021 Torres et al. 2021 Torres et al. https://creativecommons.org/licenses/by/4.0/ This content is distributed under the terms of the Creative Commons Attribution 4.0 International license.

At the start of replication, IMC32 appears as five punctae arranged in a pentagon, prior to the appearance of IMC6, suggesting that it is expressed early during endodyogeny (Fig. 1F). To more precisely analyze the timing of IMC32 expression, we colocalized IMC32 with a number of previously described proteins which also appear early during replication. We first colocalized it with ISP1, an early daughter bud marker that is incorporated into the IMC before IMC6, and found that IMC32 is recruited to the forming daughter buds before ISP1 (Fig. 1G) (21). We then colocalized IMC32 with FBXO1 and AC9, which are components of the IMC apical cap that are recruited to daughter buds at the earliest stages of endodyogeny, and found that IMC32 is recruited to early daughter buds similar to these proteins (Fig. 1H and I). Lastly, we colocalized IMC32 with the centrosome marker CEP250 and found that the early buds were adjacent to the centrosome (Fig. 1J), again consistent with IMC32 being a component of the early daughter cell scaffold (43). From this, we concluded that IMC32 is a daughter-enriched IMC protein that appears very early during parasite replication, preceding recruitment of ISP1 but at approximately the same time as AC9 and TgFBXO1.

### IMC32 is essential and plays an important role in endodyogeny.

**FIG 2:**
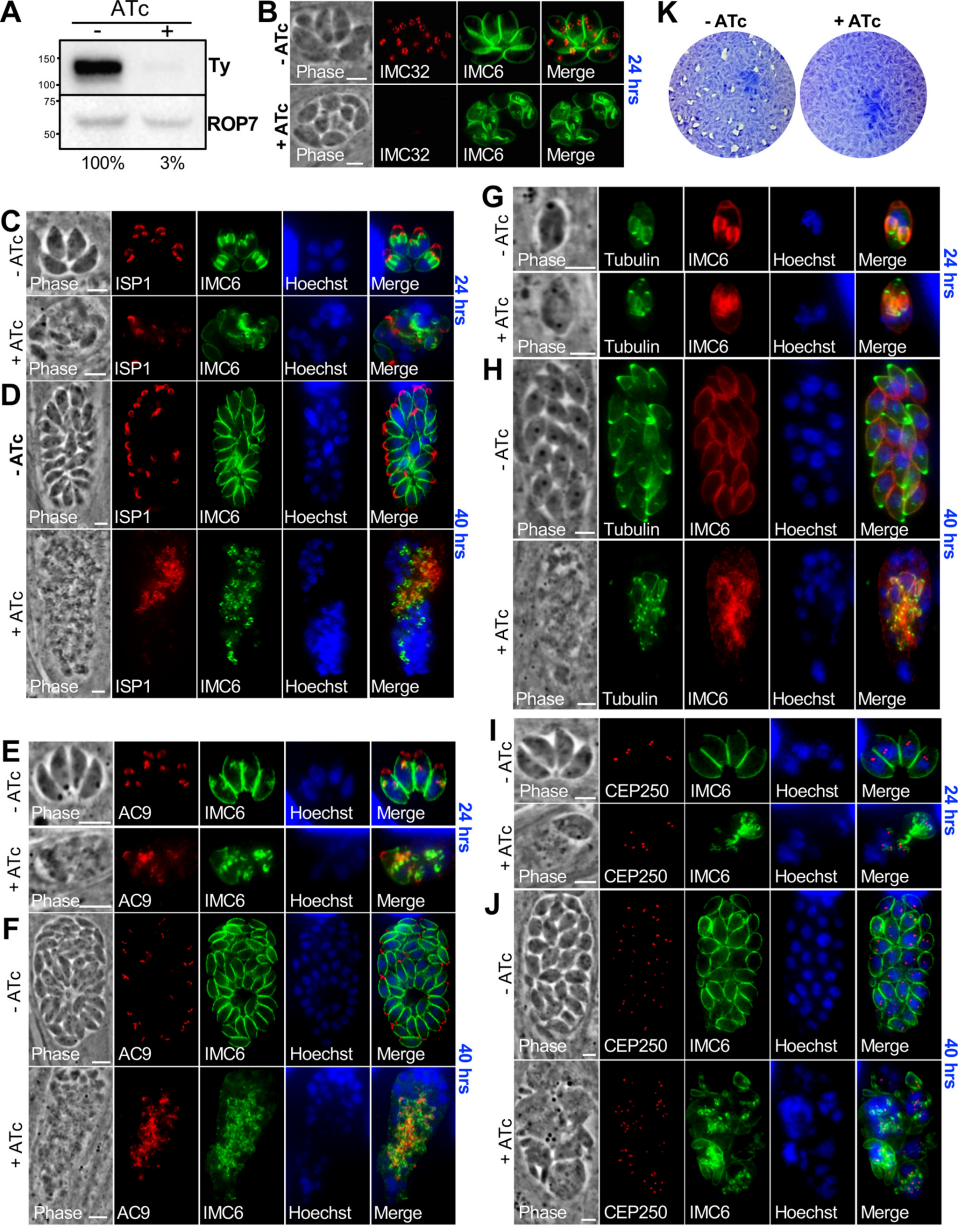
IMC32 is essential and plays an important role in endodyogeny. (A) Western blot showing ∼97% depletion of IMC32-2×Strep3×Ty after 24 h of ATc treatment. Parasites were allowed to infect for 8 h before being treated with ATc. Mouse anti-Ty was used to detect IMC32, and mouse anti-ROP7 was used as a loading control. (B) IFA of IMC32cKD knockdown with (+) or without (−) ATc showing nearly undetectable staining of IMC32 and morphological defects in the knockdown parasites. Red, mouse anti-Ty; green, rabbit anti-IMC6. (C) IFA showing IMC32cKD parasites grown with or without ATc for 24 h (following a 6-h pretreatment with or without ATc) showing aberrant morphology as seen by ISP1, IMC6, and Hoechst staining. Red, mouse anti-ISP1; green, rabbit anti-IMC6; blue, Hoechst staining. (D) Same IFA experiment as described for panel C but extended to 40 h, which shows continued attempts at replication resulting in extensive morphological defects. (E and F) IMC32cKD parasites grown with and without ATc as described for panels C and D but with staining for AC9. Green, rabbit anti-IMC6; red, mouse anti-V5 (detecting 3×V5-tagged AC9); blue, Hoechst staining. (G and H) IMC32cKD was transiently transfected with tubulin-green fluorescent protein (GFP). ATc was added following transfection, and the samples were incubated for 24 or 40 h. IFA of tubulin and IMC6 shows dramatic replication defects. Red, rabbit anti-IMC6; green, tubulin-GFP; blue, Hoechst staining. (I and J) IMC32cKD parasites grown with and without ATc as described for panels C and D but with staining for the centrosome using CEP250. Multiple centrosomes are shown in misshapen parasites. Red, mouse anti-HA; green, rabbit anti-IMC6; blue, Hoechst staining. (K) Plaque assays showing that ATc treatment eliminates the ability of IMC32cKD parasites to form plaques, demonstrating that IMC32 is essential. Scale bars for all images, 5 μm.

The negative GWCS score suggests that IMC32 is either important or essential for parasite survival (36). We were unable to disrupt the gene with CRISPR/Cas9 by using several approaches, indicating that the gene is essential. To use a conditional knockdown approach, we replaced the endogenous promoter with an anhydrotetracycline (ATc) transactivator-based regulation system, which allows for the knockdown of IMC32 transcription upon addition of ATc (IMC32cKD) (Fig. S4) (44). The promoter replacement included the introduction of an N-terminal Myc tag, which could not be detected for unknown reasons. This was not problematic, as the promoter replacement was done in a strain containing an endogenous C-terminal 2×Strep-3×Ty tag. In spite of the presumed addition of the tag and the altered timing of the truncated SAG4 promoter in the regulatable system, IMC32 localization appeared similar to that of the endogenous tagged protein (Fig. 2B). After 24 h of ATc treatment, we found by Western blotting and immunofluorescence assay (IFA) that IMC32 protein levels had decreased to almost undetectable levels (Fig. 2A and B), supporting an efficient conditional knockdown of IMC32.

10.1128/mBio.03622-20.4 FIG S4 Knockdown using Tet-regulatable promoter system. Diagram of endogenously tagged IMC32-2×Strep3×Ty, regulated by the anhydrotetracycline (ATc) transactivator-based system. In the absence of ATc, the transactivator (tTA) binds to the operator and promotes IMC32 transcription. Upon addition of ATc, tTA binds to ATc and prevents expression. Download FIG S4, TIF file, 0.1 MB. Copyright © 2021 Torres et al. 2021 Torres et al. https://creativecommons.org/licenses/by/4.0/ This content is distributed under the terms of the Creative Commons Attribution 4.0 International license.

To assess the effects of the knockdown on parasite morphology, intracellular parasites were treated for 6 h with (+) and without (−) ATc, syringe lysed, and allowed to infect new monolayers for 24 and 40 h with and without ATc. At 24 h, we saw parasites with disrupted morphology, as assessed by staining with the apical cap marker ISP1 and the cytoskeletal alveolin IMC6 (Fig. 2C). Despite these severe morphological defects, many of the vacuoles continued to expand to the 40-h time point, forming masses of misshapen parasites with a large amount of nuclear material and collapsed or missing IMC components (Fig. 2D). This suggests that in the absence of IMC32, parasites mount continued attempts at replication in spite of the gross morphological defects incurred by the knockdown. Similar results were seen by staining for tubulin and the cytoskeletal apical cap marker AC9, both of which detect very early daughter buds as well as maternal parasites (Fig. 2E to H) (28). We also examined the centrosome in these masses using CEP250 and found multiple foci, further indicating continued attempts at replication (Fig. 2I and J). The defects in replication are ultimately lethal, as the knockdown parasites fail to form plaques in a standard plaque assay (Fig. 2K). These data demonstrate that IMC32 plays a critical role in the development of the IMC and is essential for parasite viability.

### The IMC32 palmitoylation site is required for localization and function.

**FIG 3:**
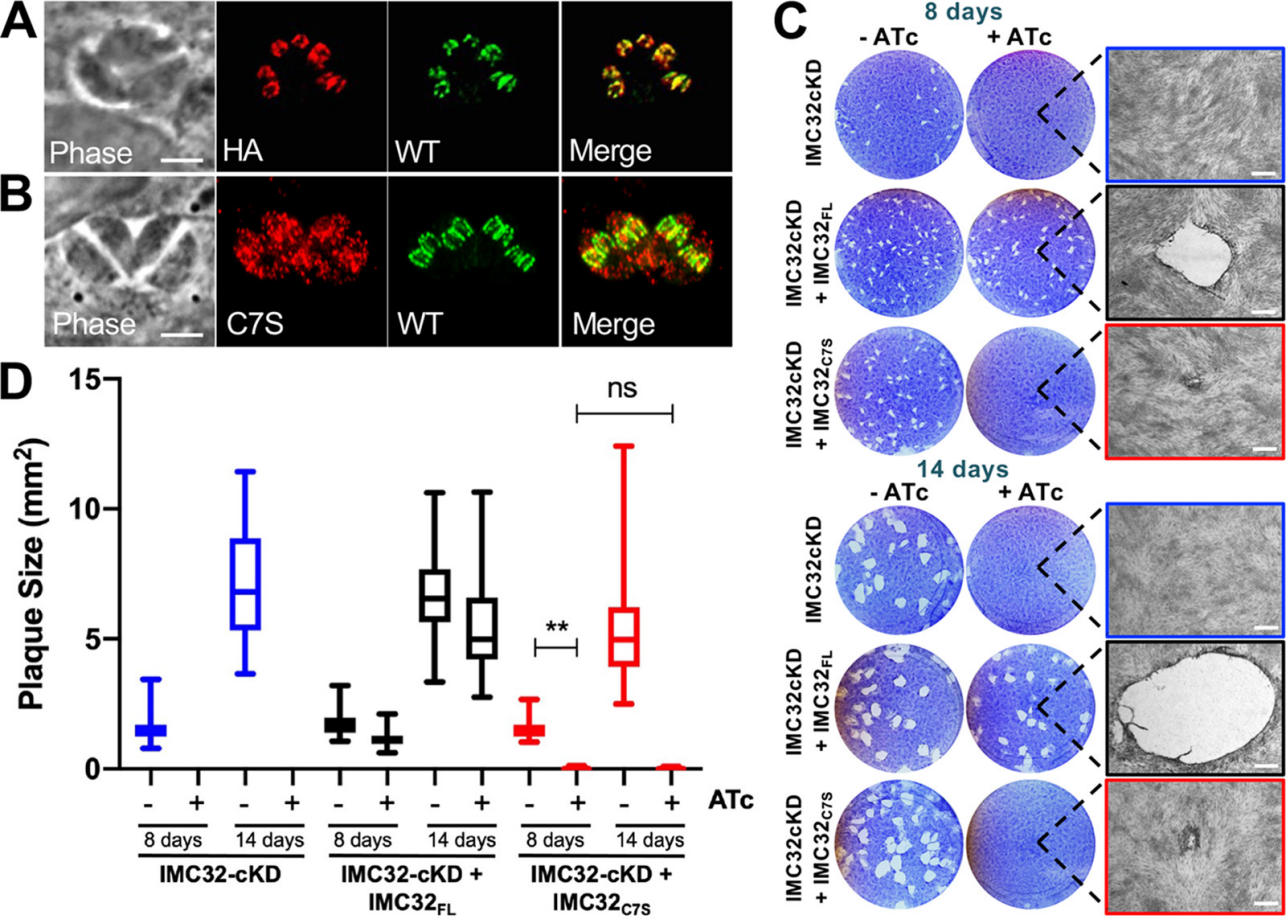
The IMC32 palmitoylation site is required for localization and function. (A) Complementation with full-length IMC32-3×HA (HA) driven from its own promoter shows colocalization with endogenous 2×Strep3×Ty-tagged IMC32 (wild type [WT]). Red, rabbit anti-HA; green, mouse anti-Ty. (B) Mutagenesis of the predicted palmitoylation site (IMC32_C7S_) results in mistargeting to punctate spots in the cytoplasm. WT, endogenous 2×Strep3×Ty-tagged IMC32. Red, rabbit anti-HA; green, mouse anti-Ty. (C) Plaque assays showing that the IMC32 knockdown cannot make plaques at 8 days postinfection. Complementation with full-length IMC32 (IMC32cKD + IMC32_FL_) rescues plaque formation, while complementation with IMC32_C7S_ results in very small plaques which do not appear larger from 8 to 14 days. Scale bar, 0.5 mm. (D) Quantification of plaque assays at days 8 and 14 showing that the small plaques formed at day 8 by IMC32_C7S_ do not grow significantly larger by extending the plaque assay (ns [not significant], *P* ≥ 0.05; **, *P* < 0.01).

To better understand how IMC32 functions in replication, we complemented the knockdown using a 3×HA-tagged wild-type cDNA driven by the endogenous promoter, targeted to the uracil phosphoribosyltransferase (UPRT) locus. The wild-type complement (IMC32cKD + IMC32_FL_) successfully colocalized with the endogenous conditionally regulated copy of IMC32 (Fig. 3A). To assess the importance of the predicted IMC32 palmitoylation site, we also generated an IMC32 expression construct in which the cysteine at residue 7 was mutated to a serine (IMC32_C7S_). Disruption of the palmitoylation site resulted in mislocalization of the protein into scattered punctae throughout the cytoplasm, showing that this residue is critical for IMC localization (Fig. 3B). We then conducted plaque assays with the three strains (IMC32cKD, IMC32cKD + IMC32_FL_, and IMC32cKD + IMC32_C7S_) and measured the mean plaque size with and without ATc. As expected, IMC32cKD parasites made large plaques without ATc and no plaques were formed upon ATc treatment (Fig. 3C and D). The IMC32cKD + IMC32_FL_ line rescued the knockdown, as it was able to form plaques that were the same size with and without ATc, noting that ATc treatment alone has a minor effect on parasite growth (Fig. S5) (44). Surprisingly, IMC32cKD + IMC32_C7S_ parasites in the presence of ATc were able to form very small plaques, with a mean plaque size only 1.7% the size of the plaques formed by IMC32cKD + IMC32_FL_ plus ATc. Extending the plaque assays to 14 days showed that the IMC32cKD + IMC32_C7S_ plaques do not get significantly larger, suggesting that these parasites fail to replicate further after forming small plaques. In agreement with this, IMC32cKD + IMC32_C7S_ parasites were unable to be cultured in the presence of ATc, and multiple attempts to knock out the endogenous wild-type copy of the gene in this background failed. Therefore, despite making small plaques initially, the IMC32_C7S_ parasites essentially cannot rescue the function of the IMC32 knockdown, demonstrating that the palmitoylation site is necessary for IMC32 localization and function.

10.1128/mBio.03622-20.5 FIG S5 Anhydrotetracycline (ATc) has a negative effect on parasite growth. Plaque assay with the parental strain of T. gondii parasites under conditions with and without ATc. There was a significant decrease (*P* < 0.01) in plaque size upon ATc treatment. Download FIG S5, TIF file, 0.2 MB. Copyright © 2021 Torres et al. 2021 Torres et al. https://creativecommons.org/licenses/by/4.0/ This content is distributed under the terms of the Creative Commons Attribution 4.0 International license.

### IMC32 localization and function are not dependent on phosphorylation.

**FIG 4:**
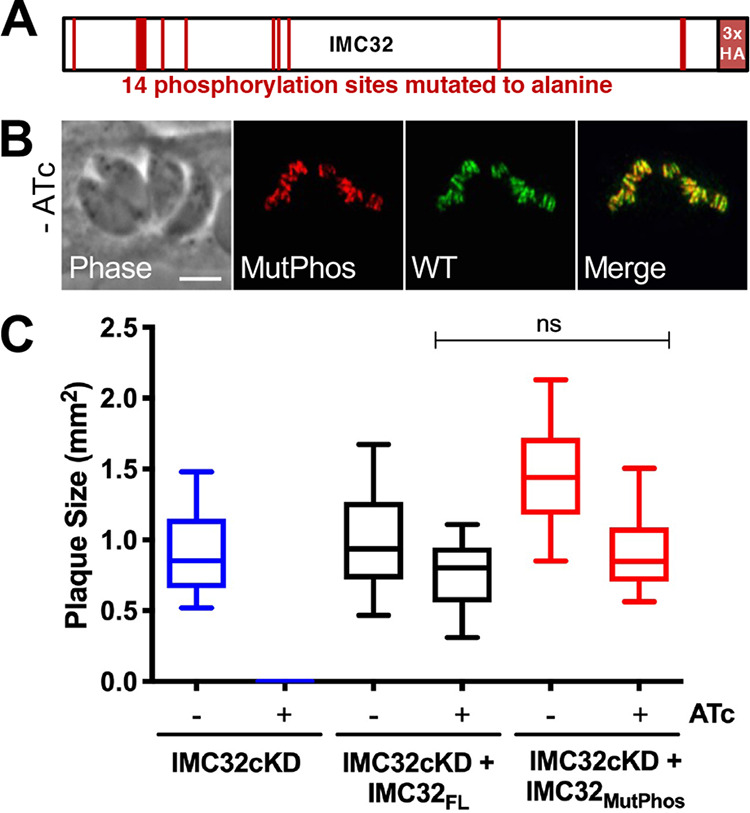
IMC32 localization and function are not dependent on phosphorylation. (A) Gene model for IMC32 with the 14 predicted phosphorylation sites mutated to alanine, plus a C-terminal 3×HA epitope tag. (B) IFA showing colocalization of endogenous IMC32 (WT) and the mutant phosphorylation copy of IMC32 expressed in the IMC32cKD + IMC32_MutPhos_ line. Red, rabbit anti-HA; green, mouse anti-Ty. Scale bar, 4 μm. (C) Quantification of plaque assays with IMC32cKD, IMC32cKD + IMC32_FL_, and IMC32cKD + IMC32_MutPhos_ parasites under conditions with and without ATc reveals no significant (*P* ≥ 0.05) difference in plaque sizes between IMC32_FL_ and IMC32_MutPhos_ with ATc.

Protein phosphorylation has been shown to play an important role in regulating the function of critical IMC components; thus, we assessed the 14 phosphorylation sites in IMC32 (Fig. 4A; Fig. S1B) (39, 45–48). To this end, we generated a construct with all 14 sites mutated to alanines and expressed it in the knockdown strain (IMC32cKD + IMC32_MutPhos_). We examined the localization of the phosphorylation mutant by IFA and found that localization was not affected (Fig. 4B). Because ATc treatment affects growth rates (Fig. S5), we compared the mutant to a strain expressing the wild-type copy of IMC32 in the presence of ATc and found no significant difference in plaque size (Fig. 4C). We therefore conclude that these phosphorylation sites are not important for the function of IMC32.

### Coiled-coil 5 is not essential for IMC32 localization or function.

Given that the phosphorylation sites were dispensable, we suspected that the five coiled-coil domains at the C terminus of the protein might play an important role in IMC32 localization or function. Coiled-coil domains are supercoils of two or more alpha-helices that are involved in protein binding-dependent functions, such as vesicle transport and structural scaffolding, which may agree with a role of IMC32 in organelle formation (49). We were particularly interested in coiled-coils 1, 4, and 5, as these regions contain substantial amino acid identity to its malarial counterpart, suggesting that they may be important for IMC32 function (Fig. S2A).

**FIG 5:**
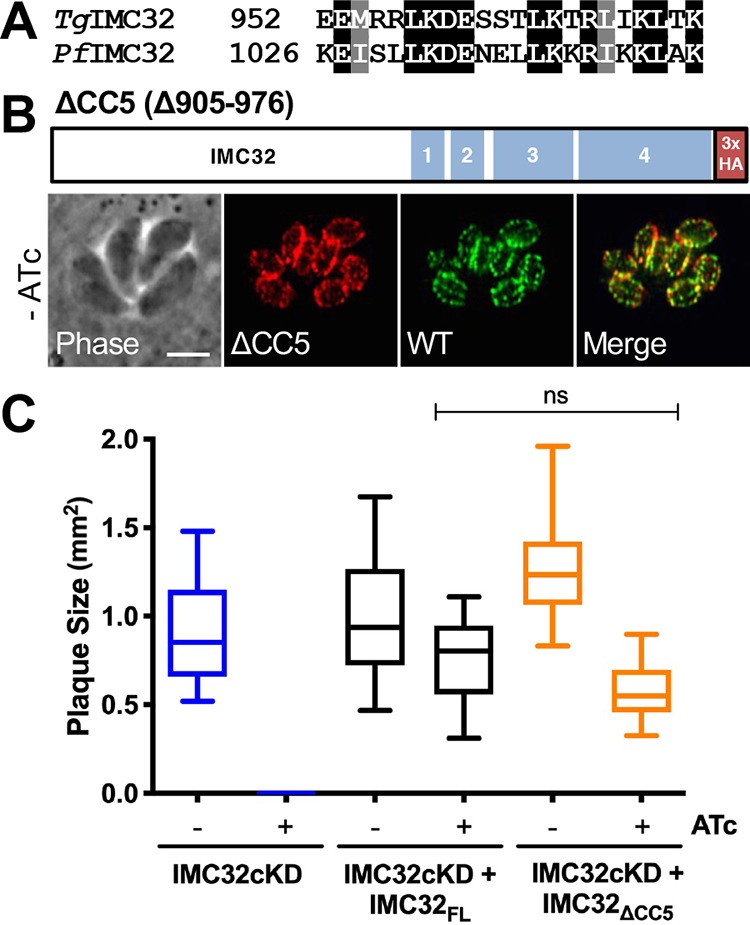
Coiled-coil 5 is not essential for IMC32 localization or function. (A) Alignment of IMC32 residues 952 to 974 with its *Plasmodium* homologue, PF3D7_0717600, showing strong similarity (full alignment available in Fig. S2 in the supplemental material). Black highlights indicate identity; gray highlights indicate similarity. (B) Diagram of IMC32_ΔCC5_. IFA shows that IMC32_ΔCC5_ localizes to daughter buds and colocalizes with 3×Ty-tagged endogenous IMC32. Red, rabbit anti-HA; green, mouse anti-Ty. Scale bar, 4 μm. (C) Plaque assay with IMC32cKD, IMC32cKD + IMC32_FL_, and IMC32cKD + IMC32_ΔCC5_ parasites under conditions with and without ATc. There was no significant difference (*P* ≥ 0.05) in plaque size between IMC32_FL_ and IMC32_ΔCC5_ with ATc.

To begin exploring these coiled-coil domains, we generated a truncated copy of IMC32 that removed just CC5 (residues 905 to 976), including a strongly conserved region in the *Plasmodium* homologue (residues 952 to 974) (Fig. 5A; Fig. S2) and asked whether the truncated protein could localize to daughter buds and effectively rescue the IMC32cKD knockdown. When expressed in the IMC32cKD line, the truncated protein (IMC32_ΔCC5_) localized correctly to the IMC (Fig. 5B). Upon knockdown of the endogenous copy, plaque assays showed that growth was not significantly reduced compared to the strain complemented with IMC32_FL_ (Fig. 5C). Thus, although part of CC5 is conserved, it is dispensable for IMC32 localization and function.

### The conserved region of coiled-coil 4 is essential for IMC32 function.

**FIG 6:**
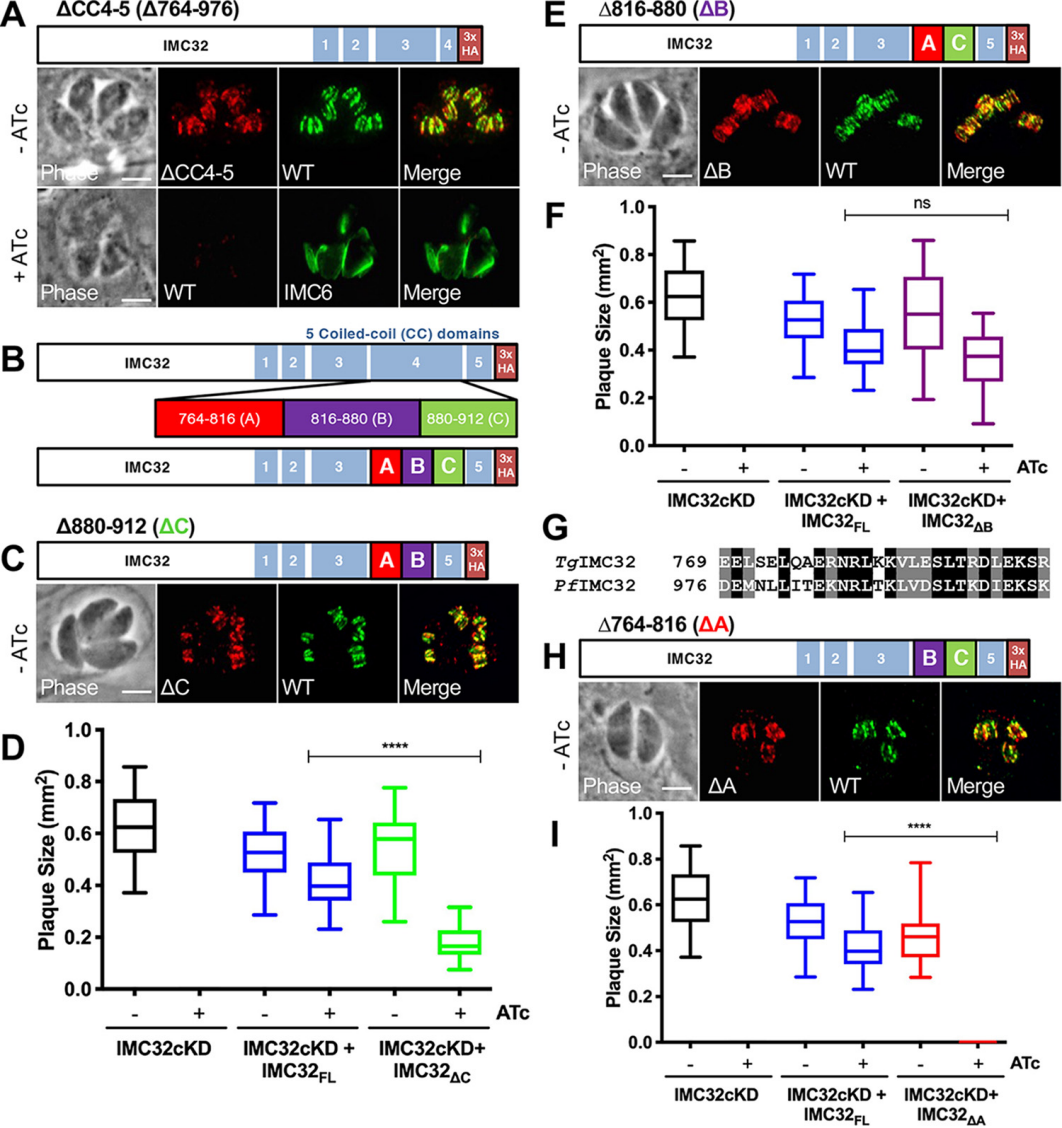
The conserved region of coiled-coil 4 is essential for IMC32 function. (A) Diagram of IMC32_ΔCC4-5_ in IMC32cKD parasites. Without ATc, IMC32_ΔCC4-5_ colocalizes with the endogenously 3×Ty-tagged IMC32 (WT). Red, rabbit anti-HA; green, mouse anti-Ty. Upon addition of ATc, endogenous IMC32 is lost, and costaining with IMC6 reveals a collapse of the IMC. Red, mouse anti-Ty; green, rabbit anti-IMC6. (B) Diagram of the three sections within coiled-coil 4 that were individually deleted for IMC32 analysis, labeled A to C. (C) Gene model of IMC32_ΔC_ and IFA showing endogenous IMC32 and IMC32_ΔC_ colocalization in daughter buds. Red, rabbit anti-HA; green, mouse anti-Ty. (D) Quantification of plaque assays with IMC32cKD, IMC32cKD + IMC32_FL_, and IMC32cKD + IMC32_ΔC_ parasites under conditions with and without ATc. The difference in plaque size between IMC32_FL_ and IMC32_ΔC_ with ATc was significant (58% reduction, *P* < 0.0001). (E) Gene model of IMC32_ΔB_ and IFA showing endogenous IMC32 and IMC32_ΔB_ colocalization in daughter buds. Red, rabbit anti-HA; green, mouse anti-Ty. (F) Quantification of plaque assays with IMC32cKD, IMC32cKD + IMC32_FL_, and IMC32cKD + IMC32_ΔB_ parasites under conditions with and without ATc. The difference in plaque size between IMC32_FL_ and IMC32_ΔB_ with ATc was not significant (*P* ≥ 0.05). (G) Alignment showing similarity of IMC32 residues 769 to 797 with its *Plasmodium* homologue, PF3D7_0717600. Black highlights indicate identity; gray highlights indicate similarity. (H) Gene model of IMC32_ΔA_ and endogenous IMC32 and IMC32_ΔA_ colocalization in daughter buds. Red, rabbit anti-HA; green, mouse anti-Ty. (I) Quantification of plaque assays with IMC32cKD, IMC32cKD + IMC32_FL_, and IMC32cKD + IMC32_ΔA_ parasites under conditions with and without ATc. IMC32_ΔA_ parasites were unable to form plaques (*P* < 0.0001). Scale bars for all images, 4μm.

To assess the role of CC4, we first removed CC5 and most of CC4 by truncating residues 764 to 976 (IMC32_ΔCC4-5_) and expressed this construct in the knockdown strain (Fig. 6A). IFA showed that IMC32_ΔCC4-5_ still localized to daughter buds (Fig. 6A, −ATc). However, we found that IMC32_ΔCC4-5_ could not complement the knockdown, as seen by morphologically disrupted parasites that phenocopy the IMC32cKD line (Fig. 6A, +ATc). This result suggests that a region within CC4 is important for IMC32 function.

To further delimit important regions of CC4, we dissected the domain in three parts, designated A to C (Fig. 6B). We then generated a series of internal deletions within CC4 and analyzed whether the deletion constructs could rescue the IMC32cKD phenotype. We first deleted region C (residues 880 to 912), which lacks homology with PF3D7_0717600. The deletion construct (IMC32_ΔC_) successfully colocalized with endogenous IMC32 in daughter buds (Fig. 6C). Upon knockdown of the endogenous copy, we observed a significant reduction in plaque size compared to IMC32_FL_ with ATc, indicating that region C of CC4 is an important but not essential region of CC4 (Fig. 6D). We next deleted region B (residues 816 to 880), which also lacks homology to PF3D7_0717600. This construct (IMC32_ΔB_) also correctly localized to the daughter buds in the IMC32cKD line and fully rescued the knockdown (Fig. 6E and F), revealing that region B is not important for IMC32 function. Finally, we assessed region A (residues 764 to 816), which contains the highest similarity between T. gondii and P. falciparum (Fig. 6G; Fig. S2). We found that IMC32_ΔA_ localizes to daughter buds but completely fails to rescue the IMC32 knockdown (Fig. 6H and I), demonstrating that this conserved region in CC4 is essential for IMC32 function.

### Coiled-coil 1 is strongly conserved and essential for protein function.

**FIG 7:**
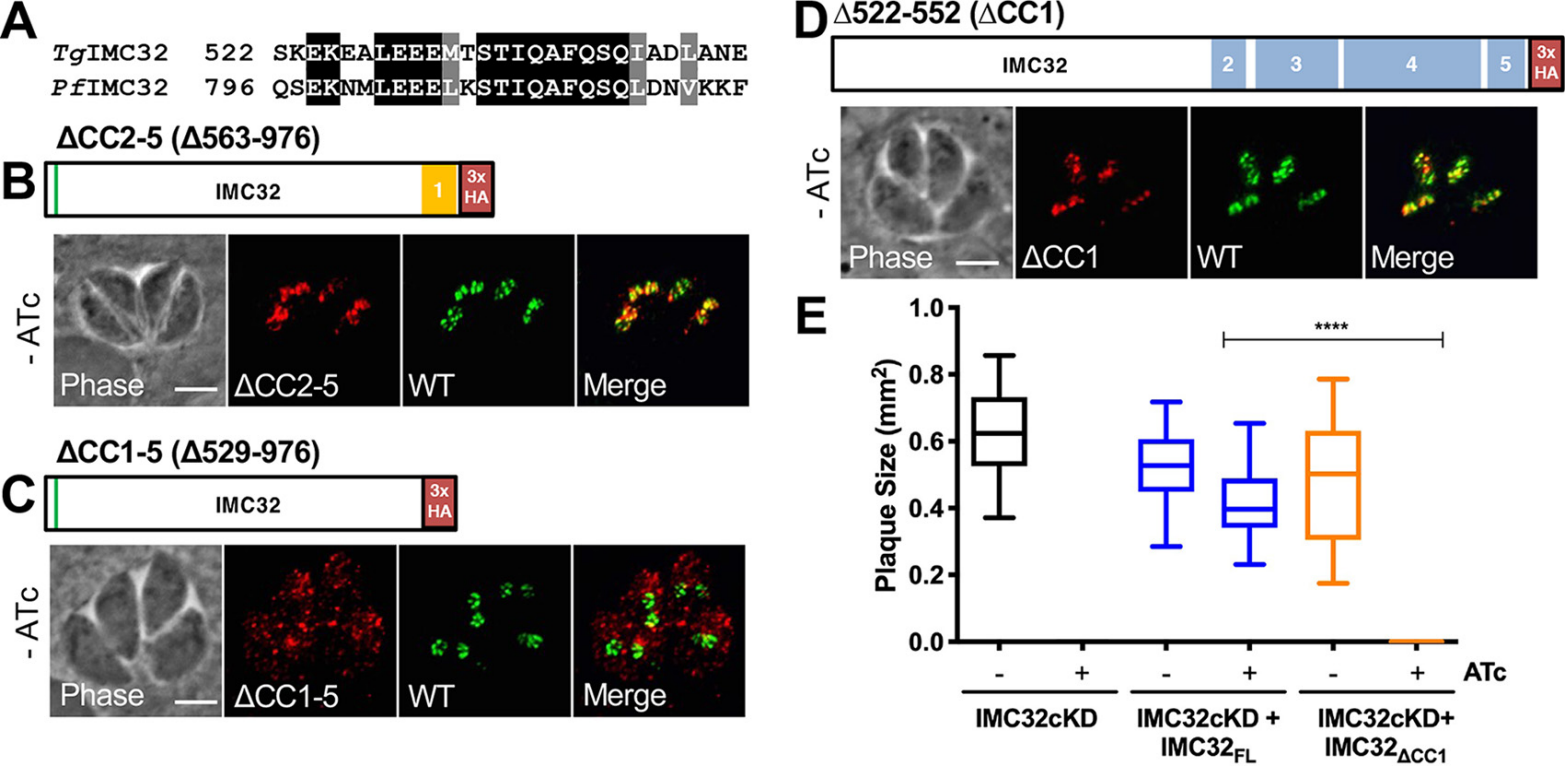
Coiled-coil 1 is strongly conserved and essential for protein function. (A) Alignment showing similarity of IMC32 residues 522 to 549 with its *Plasmodium* homologue, PF3D7_0717600. Black highlights indicate identity; gray highlights indicate similarity. (B) Gene model for IMC32_ΔCC2-5_ and localization of IMC32_ΔCC2-5_ analyzed via IFA. IMC32_ΔCC2-5_ colocalizes with the endogenous copy of IMC32 (WT) in daughter buds. Red, rabbit anti-HA; green, mouse anti-Ty. (C) Gene model for IMC32_ΔCC1-5_. IMC32_ΔCC1-5_ fails to localize to the IMC of daughter buds and instead is localized to punctae throughout the cytoplasm. Red, rabbit anti-HA; green, mouse anti-Ty. (D) Gene model of IMC32_ΔCC1_ and IFA showing IMC32_ΔCC1_ localization to daughter buds. Red, mouse anti-HA antibodies; green, Rabbit anti-Ty. (E) Quantification of plaque assays with IMC32cKD, IMC32cKD + IMC32_FL_, and IMC32cKD + IMC32_ΔCC1_ parasites under conditions with and without ATc. IMC32_ΔCC1_ parasites were unable to form plaques (*P* < 0.0001). Scale bars for all images, 4μm.

CC1 also contains a region of substantial conservation in P. falciparum (Fig. 7A; Fig. S2). Thus, we generated two additional IMC32 truncations, one which removes all of the coiled-coils except CC1 (IMC32_ΔCC2-5_) and another which truncates all of the C-terminal coiled-coil domains (IMC32_ΔCC1-5_). IMC32_ΔCC2-5_ still localized correctly with the endogenous copy of IMC32, demonstrating that the N-terminal region of the protein plus CC1 is sufficient for trafficking (Fig. 7B). Surprisingly, in spite of containing the palmitoylation site that is sufficient for localization of other IMC proteins, IMC32_ΔCC1-5_ did not localize to the daughter buds and instead localized as scattered punctae throughout the parasite (Fig. 7C). To ensure that this was not a peculiarity of this single construct, we made two other constructs to test whether the first 144 or 239 amino acids of IMC32 were sufficient for IMC targeting, and both of these showed similar spotted cytoplasmic staining (Fig. S6).

10.1128/mBio.03622-20.6 FIG S6 The N-terminal regions of IMC32 are not sufficient for IMC32 localization. (A) Diagram and IFA showing that the first 144 amino acids of IMC32 are not sufficient for IMC targeting and instead localize to cytoplasmic punctae. (B) Diagram and IFA showing that the first 239 amino acids of IMC32 are also not sufficient for IMC targeting. Red, rabbit anti-HA; green, mouse anti-Ty. Scale bar, 4 μm. Download FIG S6, TIF file, 0.5 MB. Copyright © 2021 Torres et al. 2021 Torres et al. https://creativecommons.org/licenses/by/4.0/ This content is distributed under the terms of the Creative Commons Attribution 4.0 International license.

We next generated an internal deletion which removed just CC1 and determined if this construct could localize to the daughter IMC and rescue the IMC32cKD phenotype. When analyzed by IFA, we saw that IMC32_ΔCC1_ could still target to the daughter IMC, suggesting that the remaining coils may also aid in trafficking (Fig. 7D). To assess whether CC1 was important specifically for the function of IMC32, we performed a plaque assay and found that IMC32_ΔCC1_ could not rescue the IMC32cKD phenotype (Fig. 7E). This confirmed that CC1 is essential for protein function, as suggested by its conservation with P. falciparum. Together, these data show that the palmitoylation site and at least one of the coiled-coil domains are required for trafficking to developing daughters but that the conserved CC1 is specifically essential for the function of IMC32.

## DISCUSSION

The application of BioID in T. gondii has enabled a better understanding of the protein composition of the IMC (11, 12, 28, 46). These experiments have revealed a growing number of novel proteins with unique localizations, characteristics, and functions, demonstrating the complex nature of this apicomplexan-specific organelle. We originally identified IMC32 from BioID fused to AC9, which is cytoskeletal and restricted to the apical cap, rather than membrane associated and IMC body localized like IMC32 (27, 28). Although IMC32 and AC9 reside in distinct subcompartments of the IMC, both appear very early in emerging daughter buds, suggesting roles in the initial formation of the IMC. While it is unclear whether these proteins directly interact, biotinylation of IMC32 by AC9 may occur while the proteins reside in their final destinations (which are abutting one another) or labeling may occur during the trafficking of IMC components during endodyogeny.

The tethering of IMC32 appears to be dynamic: it localizes to the IMC of daughter buds but also shows diffuse staining throughout the cytoplasm after division, rather than localizing peripherally to the maternal IMC. A similar localization is seen in the palmitoylation site mutant IMC32_C7S_, suggesting that reversible palmitoylation may allow the protein to recycle into emerging daughters at each round of replication (50). There are two palmitoyl acyltransferases in the IMC which have recently been characterized in detail, TgDHHC2 and TgDHHC14 (18). TgDHHC14 is restricted to daughter buds and is not present in the apical cap, similar to that seen for IMC32, which suggests that IMC32 may be a substrate of TgDHHC14. In addition, conditional knockdown of DHHC14 results in a similar disruption of IMC biogenesis. N-terminal acylation of other IMC proteins such as HSP20 (palmitoylation) and ISP1-3 (myristoylation and palmitoylation) is not only necessary but also sufficient for IMC membrane localization (19–22). However, we show that deletion of coiled-coils eliminates IMC32 trafficking, indicating that the palmitoylation site alone is not sufficient for localization. Although we cannot exclude the possibility that our deletion constructs (Fig. 7C; Fig. S6) caused gross misfolding of the protein, IMC32 localization appears to be dependent on both palmitoylation and its C-terminal coiled-coil domains.

While IMC32 plays a critical role in parasite replication, the precise mechanism by which it functions and how it is regulated remain to be determined. The continued expansion of the parasites upon IMC32 knockdown indicates that the parasites are not arresting in the cell cycle. Thus, the resulting phenotype is likely independent of the essential TgCyclins and TgCrk factors that serve to regulate the progression of cell division and whose disruptions result in growth arrest (6, 51). Whether or not IMC32 is regulated posttranslationally is also unknown. The simultaneous mutagenesis of all 14 predicted phosphorylation sites did not affect IMC32 function. This result does not exclude the possibility that phosphorylation of other sites might be important in regulating IMC32 or that a phosphomimetic mutant could affect its function (46, 48).

The C-terminal group of five coiled-coil domains in IMC32 suggests that this region is likely to mediate protein-protein interactions (49). Coiled-coils have been shown to be essential for GAP45, likely enabling interaction with other components of the glideosome (14). Coiled-coil domains also appear to be enriched in the apical annulus proteins that are embedded in the IMC sutures (52), as well as the conoid, which is cytoskeletal and IMC associated (53). Our dissection of the coiled-coil domains of IMC32 demonstrated that CC1 and CC4, which are most strongly conserved within P. falciparum, are essential for protein function. It is likely that these regions mediate interactions with other IMC proteins or are responsible for forming dimers or trimers of the protein. Interestingly, while CC5 is relatively well conserved and dispensable, the region just upstream of this (region C of CC4) is not conserved and necessary for optimal parasite growth. Ultimately, the identification of protein partners or structural information of the protein will provide a better understanding of how IMC32 mediates its critical role in parasite replication.

While the central functions of the IMC in motility, invasion, and replication are conserved in apicomplexans, surprisingly few of its constituents are conserved from T. gondii to P. falciparum. Conserved elements include components of the glideosome that are critical for motility and the alveolins, which make up the cytoskeletal scaffold of the organelle (8, 54, 55). In contrast, many of the membrane components appear to be unique in each organism, suggesting that these players have specialized functions within their host cells. Furthermore, most of the proteins that have been characterized are frequently expected to be dispensable based on genome-wide functional studies (11, 12, 21, 32, 36). One early daughter protein that is important for parasite replication is TgFBXO1 (32). However, TgFBXO1 localizes to the apical cap region and does not appear to be essential for parasite survival. In contrast, IMC32 localizes to the IMC body and is absolutely essential for replication, as its conditional depletion results in a lethal collapse of the IMC. Along with its early timing of expression and conservation, this suggests that IMC32 may be a foundational component of the IMC membranes during daughter bud formation. Together, this study broadens our understanding of the T. gondii IMC by revealing a conserved lynchpin of the organelle that presents a novel target for the development of therapeutics across the apicomplexan phylum.

## MATERIALS AND METHODS

### *Toxoplasma* and host cell culture.

Parental T. gondii RHΔ*hxgprt* and subsequent strains were grown on confluent monolayers of human foreskin fibroblasts (HFFs; ATCC, Manassas, VA) at 37°C and 5% CO_2_ in Dulbecco’s modified Eagle medium (DMEM) supplemented with 5% fetal bovine serum (Gibco), 5% Cosmic Calf serum (HyClone), and 1× penicillin-streptomycin-l-glutamine (Gibco). Tetracycline (Tet)-free medium for knockdown lines used 10% tetracycline-free fetal bovine serum (Omega Scientific). Constructs containing selectable markers were selected using 50 μg/ml mycophenolic acid-xanthine (HXGPRT), 40 μM chloramphenicol (CAT), or 1 μM pyrimethamine (56–58). Homologous recombination to the UPRT locus was negatively selected using 5 μM 5-fluorodeoxyuridine (59).

### Antibodies.

The hemagglutinin (HA) epitope was detected with mouse monoclonal antibody (MAb) HA.11 (BioLegend, San Diego, CA) or rabbit polyclonal antibody (pAb) anti-HA (ThermoFisher). The Ty1 epitope was detected with mouse MAb BB2 (60). Toxoplasma-specific antibodies include mouse MAb anti-ISP1 (13), rabbit pAb anti-IMC6 (61), and mouse MAb anti-ROP7 (1B10) (62).

### IFA and Western blotting.

For immunofluorescence assays (IFA), confluent HFFs grown on coverslips were infected with T. gondii. After the time periods noted in the text above, the coverslips were fixed with 3.7% formaldehyde and processed for indirect immunofluorescence as described previously (63). Primary antibodies were detected by species-specific secondary antibodies conjugated to Alexa Fluor 488/594 (ThermoFisher). Coverslips were mounted in Vectashield (Vector Labs, Burlingame, CA), viewed with an Axio Imager.Z1 fluorescence microscope (Zeiss), and processed with ZEN 2.3 software (Zeiss).

For Western blotting, intracellular parasites were lysed in 1× Laemmli sample buffer with 100 mM dithiothreitol (DTT) and boiled at 100°C for 10 min. Lysates were resolved by SDS-PAGE and transferred to nitrocellulose membranes, and proteins were detected with the appropriate primary antibody and corresponding secondary antibody conjugated to horseradish peroxidase. Chemiluminescence was induced using the SuperSignal West Pico substrate (Pierce) and imaged on a ChemiDoc XRS+ system (Bio-Rad, Hercules, CA). Quantification of Western blot signal was performed with Image Lab 5.2.1 software.

### Endogenous epitope tagging.

For C-terminal endogenous tagging, a pU6-Universal plasmid containing a protospacer against the 3′ untranslated region (UTR) of IMC32 approximately 100 bp downstream of the stop codon was generated using primers P1/P2, as described previously (41). A homology-directed repair (HDR) template was PCR amplified using the Δ*ku80*-dependent LIC vectors p3×HA.LIC-DHFR and p2×Strep-3×Ty.LIC-CAT that include the epitope tag, 3′ UTR, and selection cassette (64). The 60-bp primers (P3/P4) (Table S1) include 40 bp of homology immediately upstream of the stop codon or 40 bp of homology within the 3′ UTR downstream of the CRISPR/Cas9 cut site. This template was amplified in a total of 400 μl, purified by phenol-chloroform extraction, precipitated in ethanol, and electroporated into RH*Δhxgprt* parasites, along with 100 μg of the sequence-verified pU6-Universal plasmid. Transfected cells were allowed to invade a confluent monolayer of HFFs, and appropriate selection was applied. Successful tagging was monitored by IFA, and clonal lines of tagged parasites were obtained through limiting dilution. AC9, TgFBXO1, CEP250, and ISC6 were tagged using CRISPR/Cas9 with primers P32-P47 as described previously (12, 28, 32, 43).

10.1128/mBio.03622-20.7 TABLE S1 Oligonucleotide primers used in this study. All primer sequences are shown in the 5′ to 3′ orientation. Download Table S1, XLSX file, 0.01 MB. Copyright © 2021 Torres et al. 2021 Torres et al. https://creativecommons.org/licenses/by/4.0/ This content is distributed under the terms of the Creative Commons Attribution 4.0 International license.

### Endogenous promoter replacement.

For replacing the endogenous promoter with a Tet-regulatable one, a pU6-Universal plasmid containing a protospacer against the 5′ UTR of IMC32 approximately 100 bp upstream of the start codon was generated using primers P15/P16 (41). A homology-directed repair (HDR) template was PCR amplified using the vector pT8TATi1-HX-Tet-mycNtCOR (65), including a transactivator (tTA), seven corresponding operators (TetO), an *HXGPRT* selection cassette, and a 1×Myc tag. The 60-bp primers (P17/P18) include 40 bp of homology 100 bp upstream of the CRISPR/Cas9 cleavage site or 40 bp of homology immediately downstream of the start codon. This template was amplified in a total of 400 μl, purified by phenol-chloroform extraction, precipitated in ethanol, and electroporated into RH*Δhxgprt* parasites already endogenously tagged with 2×Strep-3×Ty-CAT, along with 100 μg of the sequence-verified pU6-Universal plasmid. Transfected cells were allowed to invade a confluent monolayer of HFFs in Tet-free medium, and appropriate selection was applied the following day. Successful tagging was monitored by IFA, and clonal lines of tagged parasites were obtained through limiting dilution.

### Complementation of IMC32cKD with IMC32HA.

The majority of the IMC32 coding region was PCR amplified from genomic DNA using primers P9/P10 and ligated together with annealed oligonucleotides P7-P8 to produce the entire coding region. This was then cloned into pUPRTKO-ISC3HA (11) using NotI/BglII (all enzymes purchased from NEB). The endogenous promoter was amplified from genomic DNA and inserted with NsiI/BglII in front of the coding sequence, resulting in pUPRTKO-IMC32HA. This complement vector was then linearized with DraIII and transfected into IMC32cKD parasites along with a universal pU6 that targets the UPRT coding region. This was followed by selection with 5 μg/ml 5-fluorodeoxyuridine for replacement of UPRT as described previously (59). IMC32HA-expressing clones were screened by IFA, and an HA-positive clone was designated IMC32_FL_.

For mutation of the predicted palmitoylation site at position 7, pUPRTKO-IMC32HA was used as a template for amplification with primers P13/P14. The resulting partial coding sequence was cloned into BglII/HpaI-digested pUPRTKO-IMC32HA. The same processes for linearization, transfection, and selection were followed as described for IMC32_FL_. Clones were screened by IFA, and an HA-positive clone was designated IMC32_C7S_. For mutagenesis of the phosphorylation sites, a synthetic gene which simultaneously changed all of the phosphorylated serines and threonines to alanine was generated.

For IMC32 C-terminal truncations, pUPRTKO-IMC32HA was used as a template to amplify truncations of the coding region. Primers P19/P20 were used for deletion of CC5 (ΔCC5), P19/P21 for ΔCC4-5, P19/P22 for ΔCC2-5, and P19/P23 for ΔCC1-5. The inserts were then cloned into a NotI-digested pUPRTKO-IMC32HA. For IMC32 internal deletions, pUPRTKO-IMC32HA was used as a template for the Q5 site-directed mutagenesis kit (NEB). Using the online NEBaseChanger tool (http://nebasechanger.neb.com/), appropriate primers were designed for each internal deletion. Primers P24/P25 were used for deletion of residues 764 to 816 (Δ764-816), P26/P27 were used for Δ816-880, P28/P29 were used for Δ880-912, and P30/P31 were used for ΔCC1. The same processes for linearization, transfection, and selection were followed for all truncation and internal deletion constructs.

### Plaque assay.

Six-well plates were seeded with HFFs and allowed to reach confluence. Serial dilutions (100, 200, and 300 parasites/well) of individual strains were used to infect the six-well plates and allowed to form plaques for 8 days, except where stated otherwise. For conditional knockdown of IMC32, ATc was added to the Tet-free medium (1 μg/ml) prior to the addition of parasites. Eight days after infection, HFF monolayers were fixed in 100% methanol for 3 min, washed with phosphate-buffered saline (PBS), and stained with crystal violet for visualization. The area of 50 plaques per condition was measured using ZEN software (Zeiss). Each plaque assay was performed a single time, because the IMC32 knockdown was lethal and we were assessing whether any level of rescue could be obtained. Statistical significance was calculated using a two-sample two-tailed *t* test. Box-and-whisker plots were generated using Prism GraphPad. The borders of the box represent the first quartile, the median, and the third quartile, while the whiskers represent minimum and maximum values.

## References

[1] Hill DE, Chirukandoth S, Dubey JP. 2005. Biology and epidemiology of Toxoplasma gondii in man and animals. Anim Health Res Rev 6:41–61. doi:10.1079/ahr2005100. 16164008

[2] Mackintosh CL, Beeson JG, Marsh K. 2004. Clinical features and pathogenesis of severe malaria. Trends Parasitol 20:597–603. doi:10.1016/j.pt.2004.09.006. 15522670

[3] Bouzid M, Hunter PR, Chalmers RM, Tyler KM. 2013. Cryptosporidium pathogenicity and virulence. Clin Microbiol Rev 26:115–134. doi:10.1128/CMR.00076-12. 23297262 PMC3553671

[4] Pappas G, Roussos N, Falagas ME. 2009. Toxoplasmosis snapshots: global status of Toxoplasma gondii seroprevalence and implications for pregnancy and congenital toxoplasmosis. Int J Parasitol 39:1385–1394. doi:10.1016/j.ijpara.2009.04.003. 19433092

[5] Gubbels M-J, Duraisingh MT. 2012. Evolution of apicomplexan secretory organelles. Int J Parasitol 42:1071–1081. doi:10.1016/j.ijpara.2012.09.009. 23068912 PMC3583008

[6] Blader IJ, Coleman BI, Chen C-T, Gubbels M-J. 2015. Lytic cycle of Toxoplasma gondii: 15 years later. Annu Rev Microbiol 69:463–485. doi:10.1146/annurev-micro-091014-104100. 26332089 PMC4659696

[7] D'Haese J, Mehlhorn H, Peters W. 1977. Comparative electron microscope study of pellicular structures in coccidia (Sarcocystis, Besnoitia and Eimeria). Int J Parasitol 7:505–518. doi:10.1016/0020-7519(77)90014-5. 413801

[8] Gould SB, Tham W-H, Cowman AF, McFadden GI, Waller RF. 2008. Alveolins, a new family of cortical proteins that define the protist infrakingdom Alveolata. Mol Biol Evol 25:1219–1230. doi:10.1093/molbev/msn070. 18359944

[9] Anderson‐White BR, Ivey FD, Cheng K, Szatanek T, Lorestani A, Beckers CJ, Ferguson DJP, Sahoo N, Gubbels M-J. 2011. A family of intermediate filament-like proteins is sequentially assembled into the cytoskeleton of Toxoplasma gondii. Cell Microbiol 13:18–31. doi:10.1111/j.1462-5822.2010.01514.x. 20698859 PMC3005026

[10] Porchet E, Torpier G. 1977. [Freeze fracture study of Toxoplasma and Sarcocystis infective stages (author’s transl)]. Z Parasitenkd 54:101–124. (In French.) doi:10.1007/BF00380795. 415447

[11] Chen AL, Kim EW, Toh JY, Vashisht AA, Rashoff AQ, Van C, Huang AS, Moon AS, Bell HN, Bentolila LA, Wohlschlegel JA, Bradley PJ. 2015. Novel components of the Toxoplasma inner membrane complex revealed by BioID. mBio 6:e02357-14. doi:10.1128/mBio.02357-14. 25691595 PMC4337574

[12] Chen AL, Moon AS, Bell HN, Huang AS, Vashisht AA, Toh JY, Lin AH, Nadipuram SM, Kim EW, Choi CP, Wohlschlegel JA, Bradley PJ. 2017. Novel insights into the composition and function of the Toxoplasma IMC sutures. Cell Microbiol 19:e12678. doi:10.1111/cmi.12678. PMC5909696 27696623

[13] Wang K, Peng ED, Huang AS, Xia D, Vermont SJ, Lentini G, Lebrun M, Wastling JM, Bradley PJ. 2016. Identification of novel O-linked glycosylated Toxoplasma proteins by Vicia villosa lectin chromatography. PLoS One 11:e0150561. doi:10.1371/journal.pone.0150561. 26950937 PMC4780768

[14] Frénal K, Polonais V, Marq J-B, Stratmann R, Limenitakis J, Soldati-Favre D. 2010. Functional dissection of the apicomplexan glideosome molecular architecture. Cell Host Microbe 8:343–357. doi:10.1016/j.chom.2010.09.002. 20951968

[15] Harding CR, Gow M, Kang JH, Shortt E, Manalis SR, Meissner M, Lourido S. 2019. Alveolar proteins stabilize cortical microtubules in Toxoplasma gondii. Nat Commun 10:401. doi:10.1038/s41467-019-08318-7. 30674885 PMC6344517

[16] Corvi MM, Berthiaume LG, De Napoli MG. 2011. Protein palmitoylation in protozoan parasites. Front Biosci (Schol Ed) 3:1067–1079. doi:10.2741/211. 21622256

[17] Alonso AM, Turowski VR, Ruiz DM, Orelo BD, Moresco JJ, Yates JR, Corvi MM. 2019. Exploring protein myristoylation in Toxoplasma gondii. Exp Parasitol 203:8–18. doi:10.1016/j.exppara.2019.05.007. 31150653 PMC6857535

[18] Dogga SK, Frénal K. 2020. Two palmitoyl acyltransferases involved sequentially in the biogenesis of the inner membrane complex of Toxoplasma gondii. Cell Microbiol 22:e13212. doi:10.1111/cmi.13212. 32329212

[19] De Napoli MG, de Miguel N, Lebrun M, Moreno SNJ, Angel SO, Corvi MM. 2013. N-terminal palmitoylation is required for Toxoplasma gondii HSP20 inner membrane complex localization. Biochim Biophys Acta 1833:1329–1337. doi:10.1016/j.bbamcr.2013.02.022. 23485398 PMC3628096

[20] Fung C, Beck JR, Robertson SD, Gubbels M-J, Bradley PJ. 2012. Toxoplasma ISP4 is a central IMC sub-compartment protein whose localization depends on palmitoylation but not myristoylation. Mol Biochem Parasitol 184:99–108. doi:10.1016/j.molbiopara.2012.05.002. 22659420 PMC3383393

[21] Beck JR, Rodriguez-Fernandez IA, de Leon JC, Huynh M-H, Carruthers VB, Morrissette NS, Bradley PJ. 2010. A novel family of Toxoplasma IMC proteins displays a hierarchical organization and functions in coordinating parasite division. PLoS Pathog 6:e1001094. doi:10.1371/journal.ppat.1001094. 20844581 PMC2936552

[22] Frénal K, Marq J-B, Jacot D, Polonais V, Soldati-Favre D. 2014. Plasticity between MyoC- and MyoA-glideosomes: an example of functional compensation in Toxoplasma gondii invasion. PLoS Pathog 10:e1004504. doi:10.1371/journal.ppat.1004504. 25393004 PMC4231161

[23] Boucher LE, Bosch J. 2015. The apicomplexan glideosome and adhesins—structures and function. J Struct Biol 190:93–114. doi:10.1016/j.jsb.2015.02.008. 25764948 PMC4417069

[24] Keeley A, Soldati D. 2004. The glideosome: a molecular machine powering motility and host-cell invasion by Apicomplexa. Trends Cell Biol 14:528–532. doi:10.1016/j.tcb.2004.08.002. 15450974

[25] Frénal K, Dubremetz J-F, Lebrun M, Soldati-Favre D. 2017. Gliding motility powers invasion and egress in Apicomplexa. Nat Rev Microbiol 15:645–660. doi:10.1038/nrmicro.2017.86. 28867819

[26] O'Shaughnessy WJ, Hu X, Beraki T, McDougal M, Reese ML. 2020. Loss of a conserved MAPK causes catastrophic failure in assembly of a specialized cilium-like structure in Toxoplasma gondii. Mol Biol Cell 31:881–888. doi:10.1091/mbc.E19-11-0607. 32073987 PMC7185968

[27] Tosetti N, Dos Santos Pacheco N, Bertiaux E, Maco B, Bournonville L, Hamel V, Guichard P, Soldati-Favre D. 2020. Essential function of the alveolin network in the subpellicular microtubules and conoid assembly in Toxoplasma gondii. Elife 9:e56635. doi:10.7554/eLife.56635. 32379047 PMC7228768

[28] Back PS, O'Shaughnessy WJ, Moon AS, Dewangan PS, Hu X, Sha J, Wohlschlegel JA, Bradley PJ, Reese ML. 2020. Ancient MAPK ERK7 is regulated by an unusual inhibitory scaffold required for Toxoplasma apical complex biogenesis. Proc Natl Acad Sci U S A 117:12164–12173. doi:10.1073/pnas.1921245117. 32409604 PMC7275706

[29] Nishi M, Hu K, Murray JM, Roos DS. 2008. Organellar dynamics during the cell cycle of Toxoplasma gondii. J Cell Sci 121:1559–1568. doi:10.1242/jcs.021089. 18411248 PMC6810632

[30] Harding CR, Meissner M. 2014. The inner membrane complex through development of Toxoplasma gondii and Plasmodium. Cell Microbiol 16:632–641. doi:10.1111/cmi.12285. 24612102 PMC4286798

[31] Striepen B, Jordan CN, Reiff S, van Dooren GG. 2007. Building the perfect parasite: cell division in Apicomplexa. PLoS Pathog 3:e78. doi:10.1371/journal.ppat.0030078. 17604449 PMC1904476

[32] Baptista CG, Lis A, Deng B, Gas-Pascual E, Dittmar A, Sigurdson W, West CM, Blader IJ. 2019. Toxoplasma F-box protein 1 is required for daughter cell scaffold function during parasite replication. PLoS Pathog 15:e1007946. doi:10.1371/journal.ppat.1007946. 31348812 PMC6685633

[33] Shaw MK, Compton HL, Roos DS, Tilney LG. 2000. Microtubules, but not actin filaments, drive daughter cell budding and cell division in Toxoplasma gondii. J Cell Sci 113:1241–1254. 10704375 10.1242/jcs.113.7.1241

[34] Dubey R, Harrison B, Dangoudoubiyam S, Bandini G, Cheng K, Kosber A, Agop-Nersesian C, Howe DK, Samuelson J, Ferguson DJP, Gubbels M-J. 2017. Differential roles for inner membrane complex proteins across Toxoplasma gondii and Sarcocystis neurona development. mSphere 2:e00409-17. doi:10.1128/mSphere.00409-17. 29062899 PMC5646244

[35] Behnke MS, Wootton JC, Lehmann MM, Radke JB, Lucas O, Nawas J, Sibley LD, White MW. 2010. Coordinated progression through two subtranscriptomes underlies the tachyzoite cycle of Toxoplasma gondii. PLoS One 5:e12354. doi:10.1371/journal.pone.0012354. 20865045 PMC2928733

[36] Sidik SM, Huet D, Ganesan SM, Huynh M-H, Wang T, Nasamu AS, Thiru P, Saeij JPJ, Carruthers VB, Niles JC, Lourido S. 2016. A genome-wide CRISPR screen in Toxoplasma identifies essential apicomplexan genes. Cell 166:1423–1435.e12. doi:10.1016/j.cell.2016.08.019. 27594426 PMC5017925

[37] Ren J, Wen L, Gao X, Jin C, Xue Y, Yao X. 2008. CSS-Palm 2.0: an updated software for palmitoylation sites prediction. Protein Eng Des Sel 21:639–644. doi:10.1093/protein/gzn039. 18753194 PMC2569006

[38] Lupas A, Dyke MV, Stock J. 1991. Predicting coiled coils from protein sequences. Science 252:1162–1164. doi:10.1126/science.252.5009.1162. 2031185

[39] Treeck M, Sanders JL, Elias JE, Boothroyd JC. 2011. The phosphoproteomes of Plasmodium falciparum and Toxoplasma gondii reveal unusual adaptations within and beyond the parasites’ boundaries. Cell Host Microbe 10:410–419. doi:10.1016/j.chom.2011.09.004. 22018241 PMC3254672

[40] Zhang M, Wang C, Otto TD, Oberstaller J, Liao X, Adapa SR, Udenze K, Bronner IF, Casandra D, Mayho M, Brown J, Li S, Swanson J, Rayner JC, Jiang RHY, Adams JH. 2018. Uncovering the essential genes of the human malaria parasite Plasmodium falciparum by saturation mutagenesis. Science 360:eaap7847. doi:10.1126/science.aap7847. 29724925 PMC6360947

[41] Sidik SM, Hackett CG, Tran F, Westwood NJ, Lourido S. 2014. Efficient genome engineering of Toxoplasma gondii using CRISPR/Cas9. PLoS One 9:e100450. doi:10.1371/journal.pone.0100450. 24971596 PMC4074098

[42] Viswanathan S, Williams ME, Bloss EB, Stasevich TJ, Speer CM, Nern A, Pfeiffer BD, Hooks BM, Li W-P, English BP, Tian T, Henry GL, Macklin JJ, Patel R, Gerfen CR, Zhuang X, Wang Y, Rubin GM, Looger LL. 2015. High-performance probes for light and electron microscopy. Nat Methods 12:568–576. doi:10.1038/nmeth.3365. 25915120 PMC4573404

[43] Suvorova ES, Francia M, Striepen B, White MW. 2015. A novel bipartite centrosome coordinates the apicomplexan cell cycle. PLoS Biol 13:e1002093. doi:10.1371/journal.pbio.1002093. 25734885 PMC4348508

[44] Meissner M, Brecht S, Bujard H, Soldati D. 2001. Modulation of myosin A expression by a newly established tetracycline repressor-based inducible system in Toxoplasma gondii. Nucleic Acids Res 29:e115. doi:10.1093/nar/29.22.e115. 11713335 PMC92585

[45] Dogga SK, Mukherjee B, Jacot D, Kockmann T, Molino L, Hammoudi P-M, Hartkoorn RC, Hehl AB, Soldati-Favre D. 2017. A druggable secretory protein maturase of Toxoplasma essential for invasion and egress. Elife 6:e27480. doi:10.7554/eLife.27480. 28898199 PMC5595437

[46] Gaji RY, Johnson DE, Treeck M, Wang M, Hudmon A, Arrizabalaga G. 2015. Phosphorylation of a myosin motor by TgCDPK3 facilitates rapid initiation of motility during Toxoplasma gondii egress. PLoS Pathog 11:e1005268. doi:10.1371/journal.ppat.1005268. 26544049 PMC4636360

[47] Gilk SD, Gaskins E, Ward GE, Beckers CJM. 2009. GAP45 phosphorylation controls assembly of the Toxoplasma myosin XIV complex. Eukaryot Cell 8:190–196. doi:10.1128/EC.00201-08. 19047362 PMC2643604

[48] Wallbank BA, Dominicus CS, Broncel M, Legrave N, Kelly G, MacRae JI, Staines HM, Treeck M. 2019. Characterisation of the Toxoplasma gondii tyrosine transporter and its phosphorylation by the calcium‐dependent protein kinase 3. Mol Microbiol 111:1167–1181. doi:10.1111/mmi.14156. 30402958 PMC6488386

[49] Lupas AN, Bassler J. 2017. Coiled coils—a model system for the 21st century. Trends Biochem Sci 42:130–140. doi:10.1016/j.tibs.2016.10.007. 27884598

[50] Mumby SM. 1997. Reversible palmitoylation of signaling proteins. Curr Opin Cell Biol 9:148–154. doi:10.1016/s0955-0674(97)80056-7. 9069258

[51] Alvarez CA, Suvorova ES. 2017. Checkpoints of apicomplexan cell division identified in Toxoplasma gondii. PLoS Pathog 13:e1006483. doi:10.1371/journal.ppat.1006483. 28671988 PMC5510908

[52] Engelberg K, Chen C-T, Bechtel T, Guzmán VS, Drozda AA, Chavan S, Weerapana E, Gubbels M-J. 2020. The apical annuli of Toxoplasma gondii are composed of coiled-coil and signalling proteins embedded in the inner membrane complex sutures. Cell Microbiol 22:e13112. doi:10.1111/cmi.13112. 31470470 PMC6925623

[53] Graindorge A, Frénal K, Jacot D, Salamun J, Marq JB, Soldati-Favre D. 2016. The conoid associated motor MyoH is indispensable for Toxoplasma gondii entry and exit from host cells. PLoS Pathog 12:e1005388. doi:10.1371/journal.ppat.1005388. 26760042 PMC4711953

[54] Baum J, Richard D, Healer J, Rug M, Krnajski Z, Gilberger T-W, Green JL, Holder AA, Cowman AF. 2006. A conserved molecular motor drives cell invasion and gliding motility across malaria life cycle stages and other apicomplexan parasites. J Biol Chem 281:5197–5208. doi:10.1074/jbc.M509807200. 16321976

[55] Al-Khattaf FS, Tremp AZ, Dessens JT. 2015. Plasmodium alveolins possess distinct but structurally and functionally related multi-repeat domains. Parasitol Res 114:631–639. doi:10.1007/s00436-014-4226-9. 25475193 PMC4303705

[56] Donald RGK, Carter D, Ullman B, Roos DS. 1996. Insertional tagging, cloning, and expression of the Toxoplasma gondii hypoxanthine-xanthine-guanine phosphoribosyltransferase gene. Use as a selectable marker for stable transformation. J Biol Chem 271:14010–14019. doi:10.1074/jbc.271.24.14010. 8662859

[57] Donald RG, Roos DS. 1993. Stable molecular transformation of Toxoplasma gondii: a selectable dihydrofolate reductase-thymidylate synthase marker based on drug-resistance mutations in malaria. Proc Natl Acad Sci U S A 90:11703–11707. doi:10.1073/pnas.90.24.11703. 8265612 PMC48052

[58] Kim K, Soldati D, Boothroyd JC. 1993. Gene replacement in Toxoplasma gondii with chloramphenicol acetyltransferase as selectable marker. Science 262:911–914. doi:10.1126/science.8235614. 8235614

[59] Donald RG, Roos DS. 1995. Insertional mutagenesis and marker rescue in a protozoan parasite: cloning of the uracil phosphoribosyltransferase locus from Toxoplasma gondii. Proc Natl Acad Sci U S A 92:5749–5753. doi:10.1073/pnas.92.12.5749. 7777580 PMC41774

[60] Bastin P, Bagherzadeh A, Matthews KR, Gull K. 1996. A novel epitope tag system to study protein targeting and organelle biogenesis in Trypanosoma brucei. Mol Biochem Parasitol 77:235–239. doi:10.1016/0166-6851(96)02598-4. 8813669

[61] Choi CP, Moon AS, Back PS, Jami‐Alahmadi Y, Vashisht AA, Wohlschlegel JA, Bradley PJ. 2019. A photoactivatable crosslinking system reveals protein interactions in the Toxoplasma gondii inner membrane complex. PLoS Biol 17:e3000475. doi:10.1371/journal.pbio.3000475. 31584943 PMC6795473

[62] Rome ME, Beck JR, Turetzky JM, Webster P, Bradley PJ. 2008. Intervacuolar transport and unique topology of GRA14, a novel dense granule protein in Toxoplasma gondii. Infect Immun 76:4865–4875. doi:10.1128/IAI.00782-08. 18765740 PMC2573327

[63] Bradley PJ, Ward C, Cheng SJ, Alexander DL, Coller S, Coombs GH, Dunn JD, Ferguson DJ, Sanderson SJ, Wastling JM, Boothroyd JC. 2005. Proteomic analysis of rhoptry organelles reveals many novel constituents for host-parasite interactions in Toxoplasma gondii. J Biol Chem 280:34245–34258. doi:10.1074/jbc.M504158200. 16002398

[64] Huynh M-H, Carruthers VB. 2009. Tagging of endogenous genes in a Toxoplasma gondii strain lacking Ku80. Eukaryot Cell 8:530–539. doi:10.1128/EC.00358-08. 19218426 PMC2669203

[65] Meissner M, Krejany E, Gilson PR, de Koning-Ward TF, Soldati D, Crabb BS. 2005. Tetracycline analogue-regulated transgene expression in Plasmodium falciparum blood stages using Toxoplasma gondii transactivators. Proc Natl Acad Sci U S A 102:2980–2985. doi:10.1073/pnas.0500112102. 15710888 PMC548799

[66] Madeira F, Mi Park Y, Lee J, Buso N, Gur T, Madhusoodanan N, Basutkar P, Tivey ARN, Potter SC, Finn RD, Lopez R. 2019. The EMBL-EBI search and sequence analysis tools APIs in 2019. Nucleic Acids Res 47:W636–W641. doi:10.1093/nar/gkz268. 30976793 PMC6602479

