# Supplementary figures and images for: Identification and Molecular Dissection of IMC32, a Conserved Toxoplasma Inner Membrane Complex Protein That Is Essential for Parasite Replication
Source: mBio. 2021 Feb 16;12(1):e03622-20. doi: 10.1128/mBio.03622-20 (PMC8545131; doi:10.1128/mBio.03622-20)

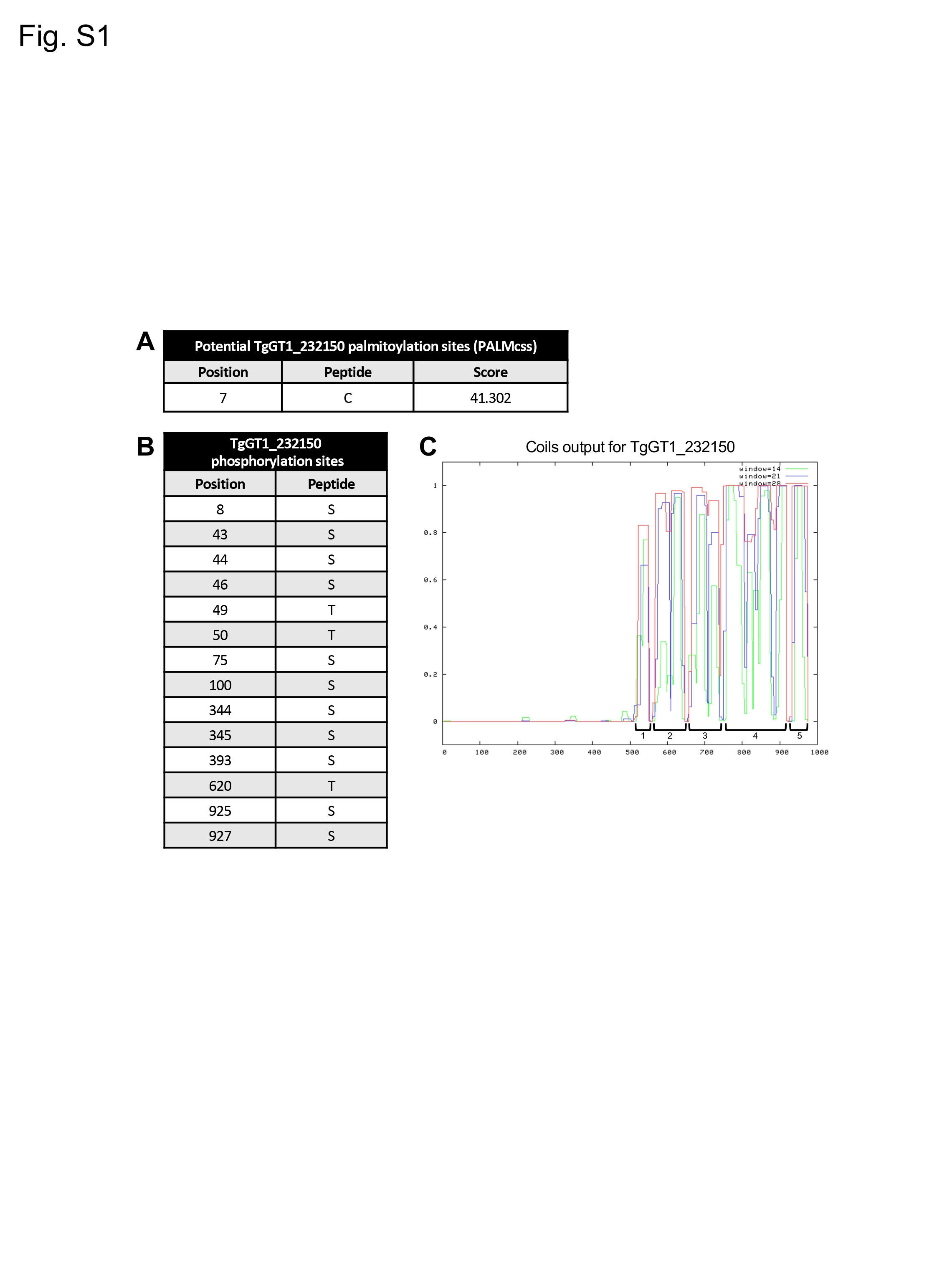

Supplement: FIG S1 [file mbio.03622-20-sf001.tif]

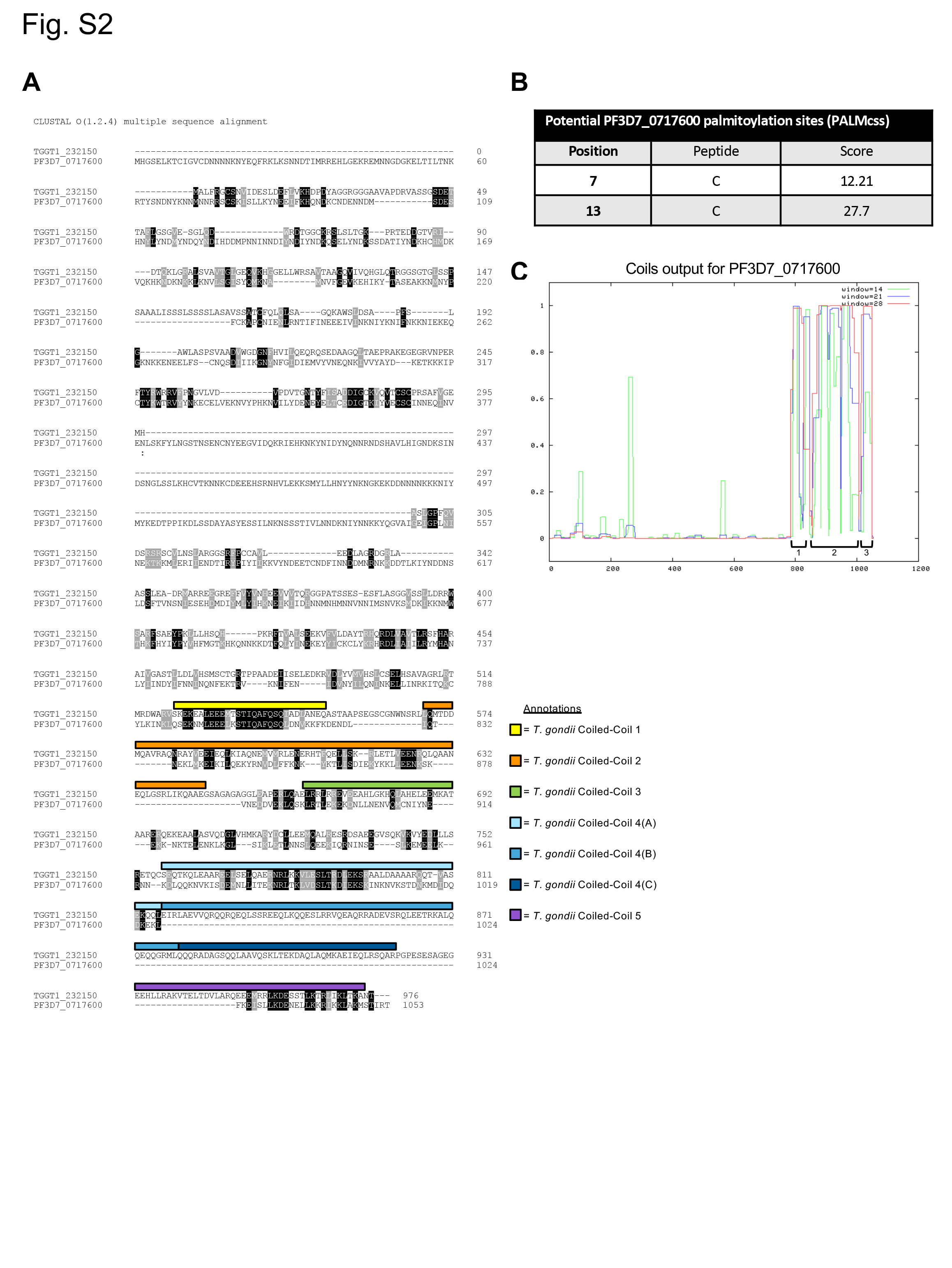

Supplement: FIG S2 [file mbio.03622-20-sf002.tif]

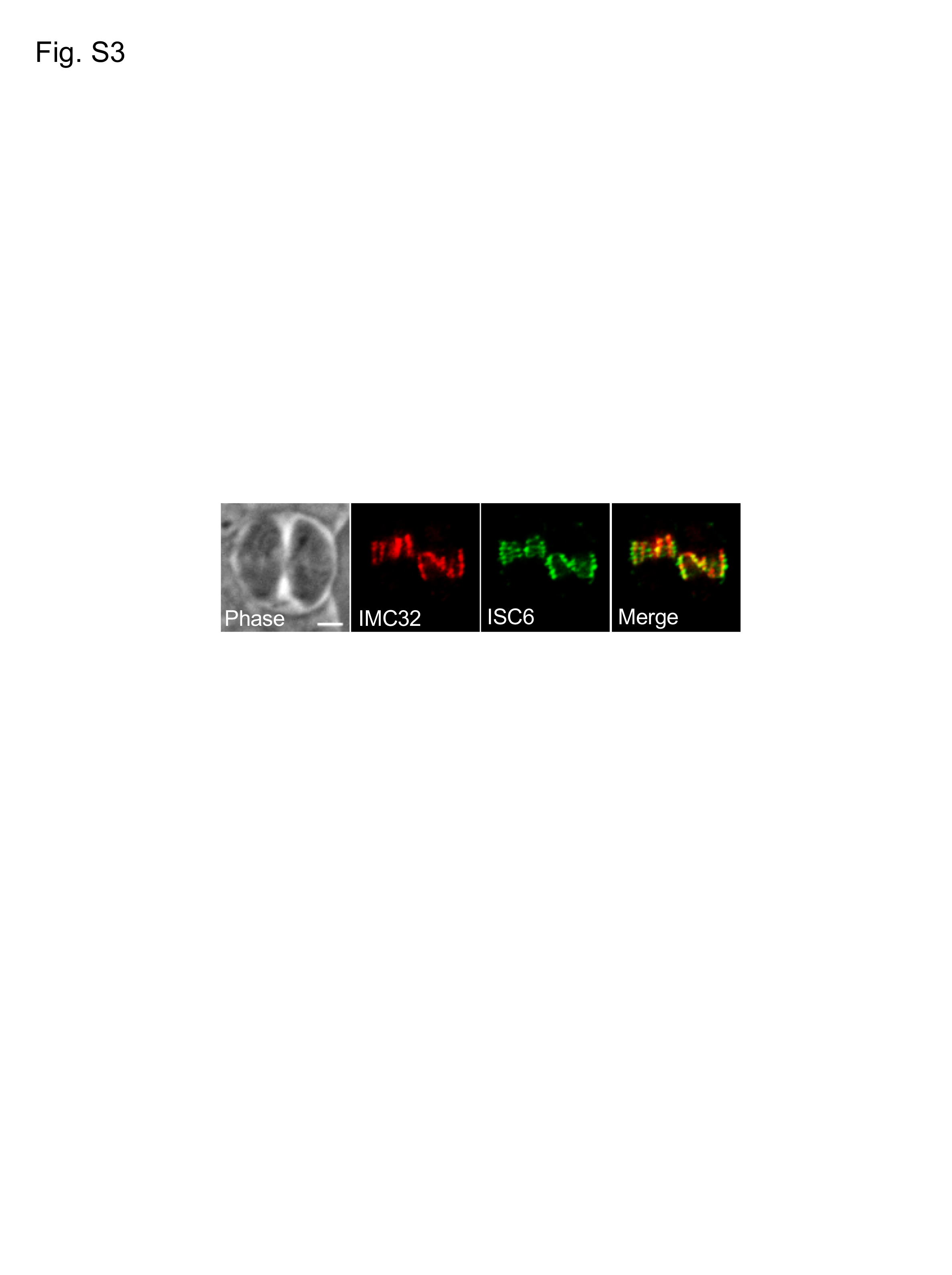

Supplement: FIG S3 [file mbio.03622-20-sf003.tif]

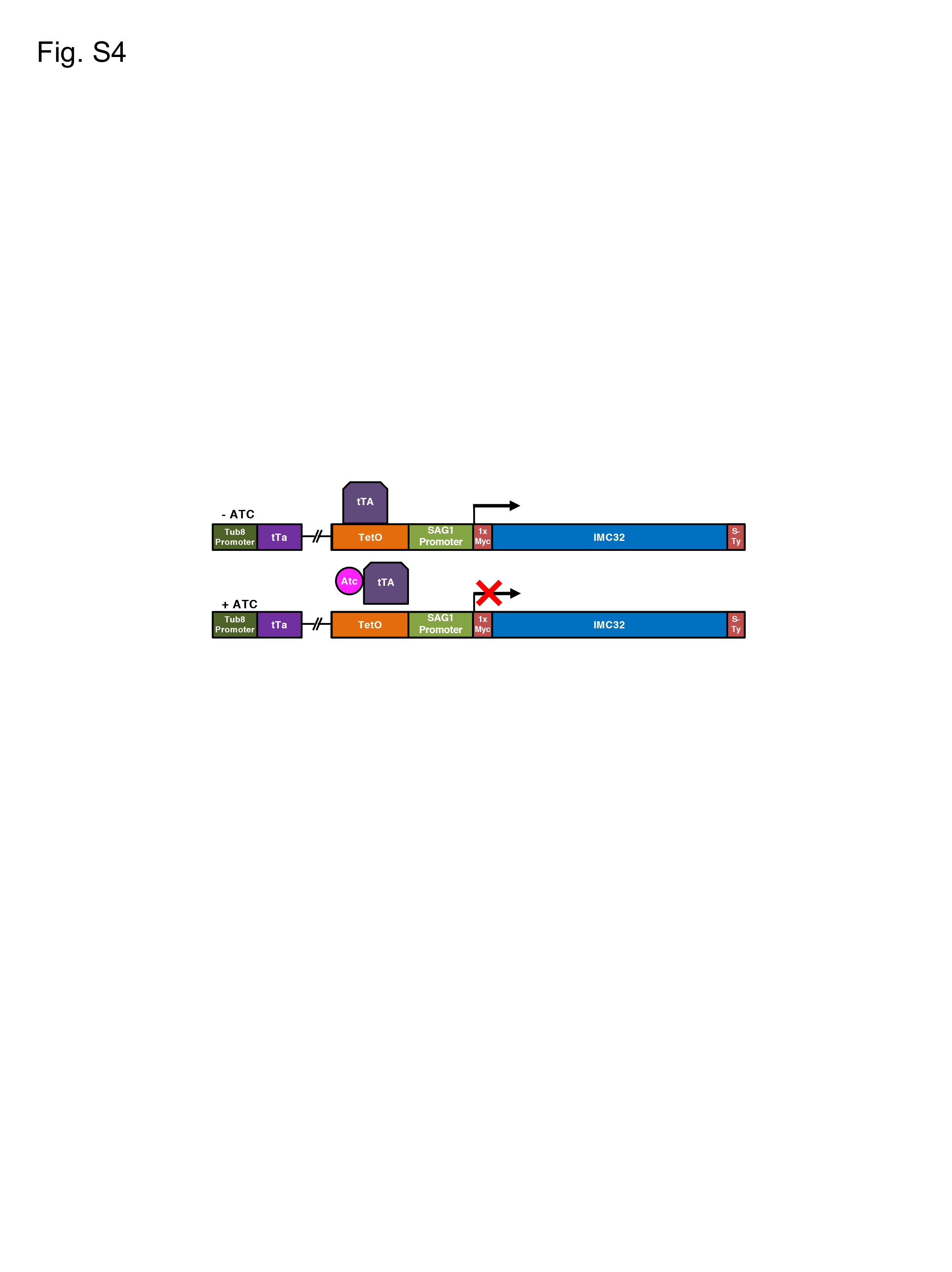

Supplement: FIG S4 [file mbio.03622-20-sf004.tif]

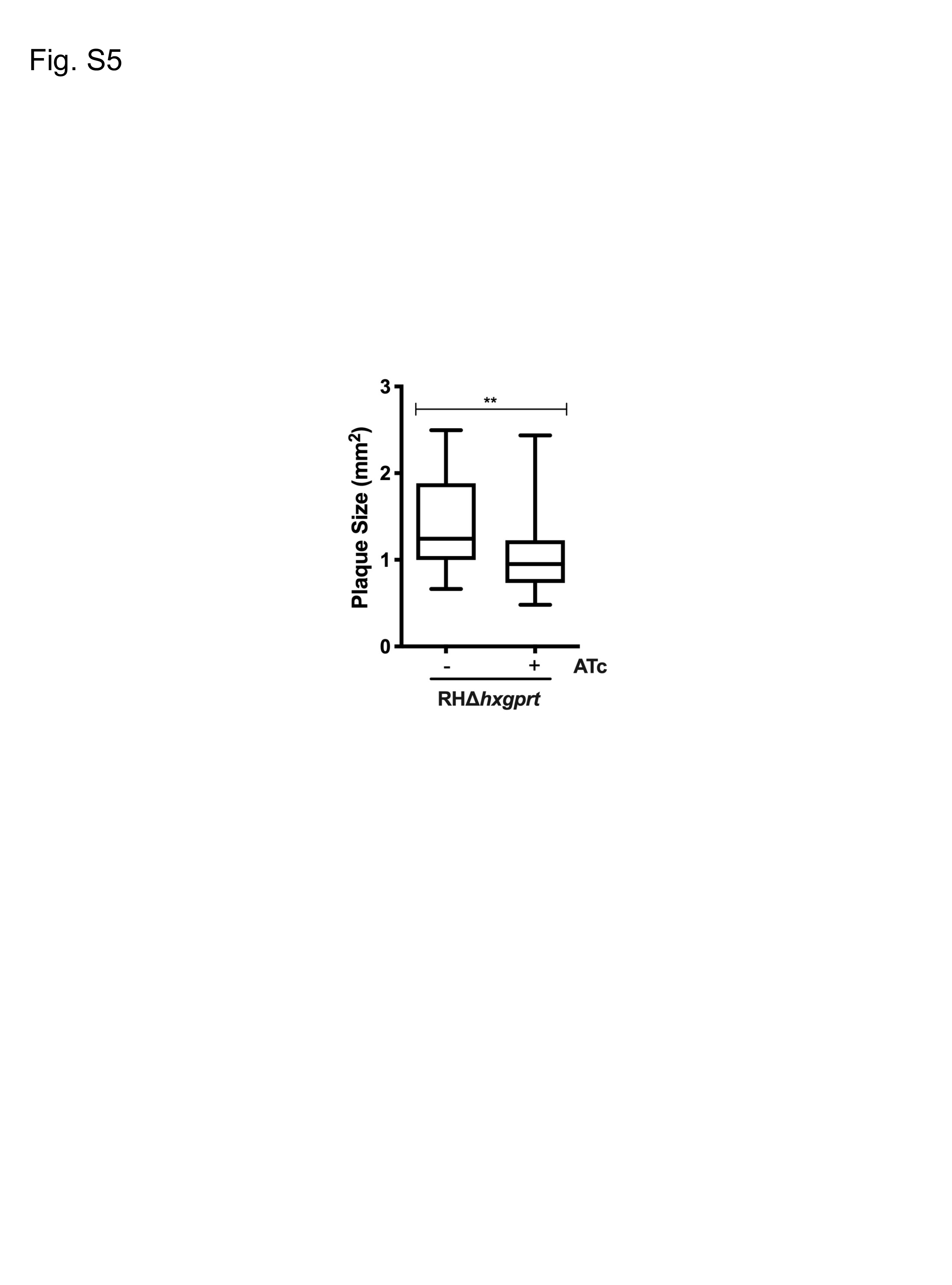

Supplement: FIG S5 [file mbio.03622-20-sf005.tif]

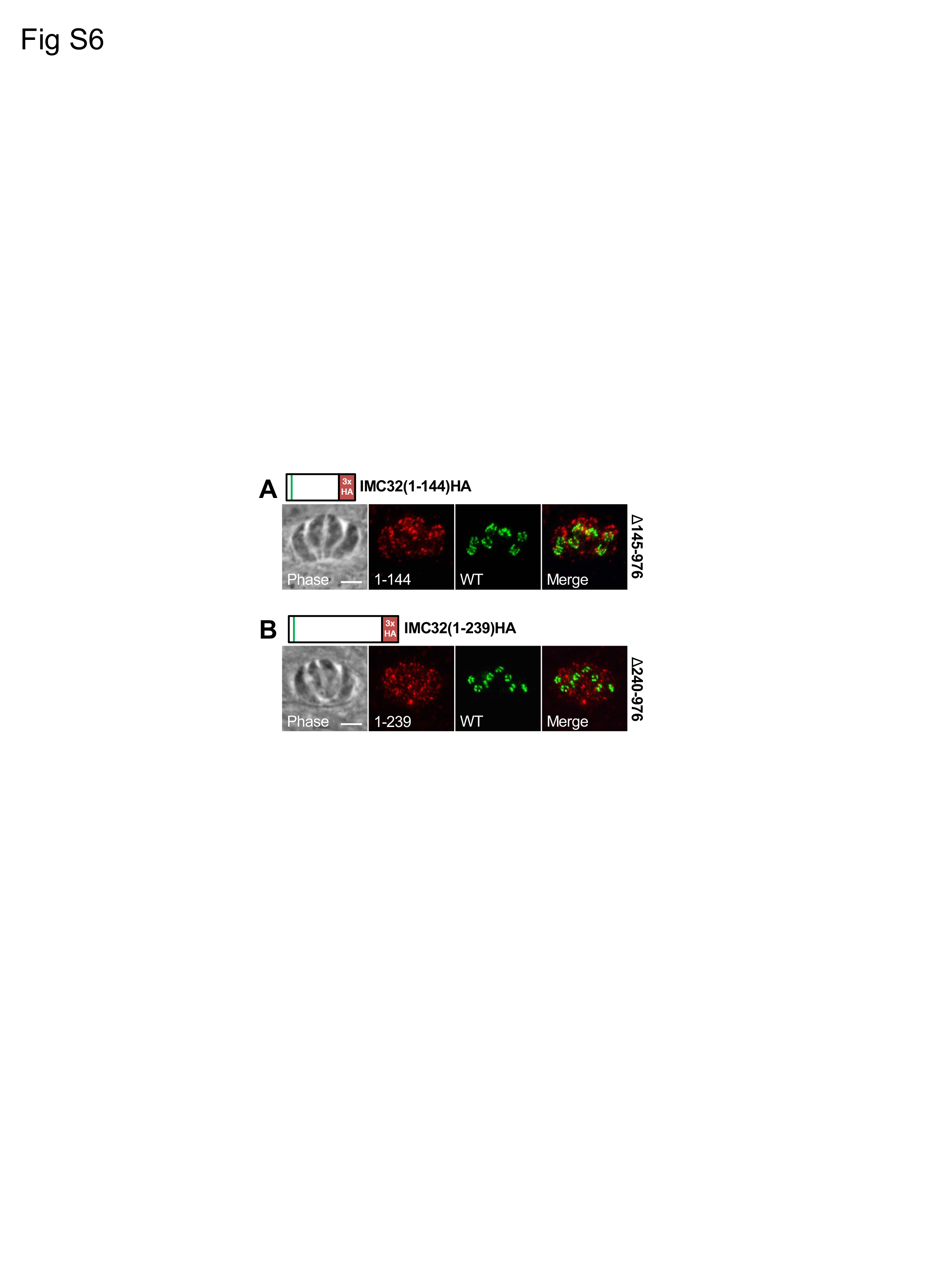

Supplement: FIG S6 [file mbio.03622-20-sf006.tif]
